# Machine learning in chemical reaction space

**DOI:** 10.1038/s41467-020-19267-x

**Published:** 2020-10-30

**Authors:** Sina Stocker, Gábor Csányi, Karsten Reuter, Johannes T. Margraf

**Affiliations:** 1 Chair of Theoretical Chemistry and Catalysis Research Center, Technische Universität München, Garching, Germany; 2 Engineering Laboratory, University of Cambridge, Cambridge, CB2 1PZ UK; 3 Fritz-Haber-Institut der Max-Planck-Gesellschaft, Berlin, Germany

**Keywords:** Catalysis, Physical chemistry, Computational chemistry, Density functional theory, Method development

## Abstract

Chemical compound space refers to the vast set of all possible chemical compounds, estimated to contain 10^60^ molecules. While intractable as a whole, modern machine learning (ML) is increasingly capable of accurately predicting molecular properties in important subsets. Here, we therefore engage in the ML-driven study of even larger reaction space. Central to chemistry as a science of transformations, this space contains all possible chemical reactions. As an important basis for ‘reactive’ ML, we establish a first-principles database (Rad-6) containing closed and open-shell organic molecules, along with an associated database of chemical reaction energies (Rad-6-RE). We show that the special topology of reaction spaces, with central hub molecules involved in multiple reactions, requires a modification of existing compound space ML-concepts. Showcased by the application to methane combustion, we demonstrate that the learned reaction energies offer a non-empirical route to rationally extract reduced reaction networks for detailed microkinetic analyses.

## Abstract (web summary)

Application of machine-learning approaches to exploring chemical reaction networks is challenging due to need of including open-shell reaction intermediates. Here the authors introduce a density functional theory database of closed and open-shell molecules for machine-learning predictions of reaction energies.

## Introduction

Reaction networks are essential tools for the description, illustration, and fundamental understanding of chemical processes in such diverse fields as catalysis^1–4^, combustion^5–7^, polymerization^8^, atmospheric chemistry^9^, systems chemistry^10,11^, and the origin of life^12^. Indeed, any study of chemical kinetics or selectivity is essentially a study of a reaction network. In many cases, however, the understanding of complex chemical processes is hampered by the sheer size of the networks in question^1,13–21^. For example, we recently reported a database of over 1 million elementary reactions for molecules no larger than four non-hydrogen atoms containing carbon, oxygen and hydrogen^22^.

**Fig. 1:**
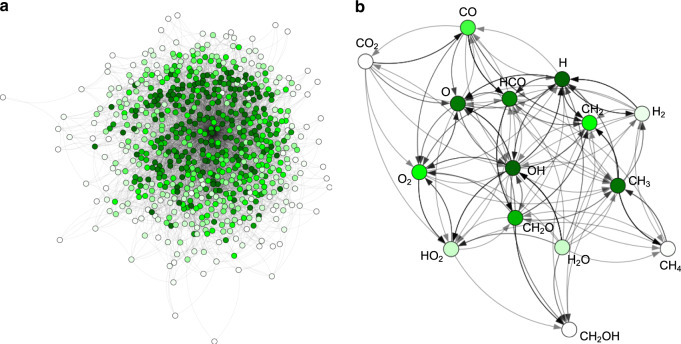
Visualization of chemical reaction spaces as graphs with molecules as nodes and reactions as edges. **a** Full network of bond dissociation reactions for carbon-, oxygen-, hydrogen-containing molecules with up to four heavy atoms. **b** Reduced reaction network of the initial steps of natural gas combustion. Nodes are colored according to the number of incident edges/reactions (their degree) from low (white) to high (dark green).

The reaction networks typically used in microkinetic studies of natural and industrial processes are therefore necessarily merely sub-graphs of the full network of possible reactions (see Fig. 1)^20,23^. This is not automatically a problem, as large parts of the latter may not be thermodynamically accessible. It is therefore entirely possible that a microkinetic model based on a reduced reaction network correctly describes the overall kinetics of a complex process^1,6,20^. Meanwhile, the big advantage of focusing on sub-graphs is that the kinetics and thermochemistry of each elementary step may be explicitly computed from first principles. This offers a non-empirical route to understanding complex reaction mechanisms.

Notwithstanding, the difficulty lies in knowing which parts of the full network to keep. One would need at least an approximate notion of the reaction thermochemistry (and ideally the kinetics) of the full network, to be able to do this on a rational basis. This information is typically not available. Indeed, not even the topology of the full network is usually taken into account. Instead, state-of-the-art reaction networks are generally built by hand, based on chemical intuition and (sparse) experimental evidence. The frequently observed failure to correctly predict the selectivities of complex catalytic processes with first–principles microkinetics indicates that such ad hoc networks may miss important links^24–26^.

The central impediment towards a non-empirical construction of reduced reaction networks is the large computational cost of first-principles electronic structure methods such as density-functional theory (DFT). It is simply not feasible to routinely compute tens or hundreds of thousands of reaction energies (REs) and activation barriers. In this context, machine-learning (ML) models that are trained on a limited number of DFT calculations have recently emerged as powerful tools for the high-throughput prediction of molecular and materials properties^27–33^. Simply put, ML can be used to interpolate properties (such as energies) across chemical compound space. State-of-the-art methods actually surpass chemical accuracy (ca. 0.05 eV) when applied to standard benchmarks like the QM9 database^34–38^. Similarly, ML models can be applied to conformational space (e.g., when trained on ab initio molecular dynamics trajectories) or even interpolate across chemical and conformational space at the same time^39–41^.

While exploring compound space is useful in its own right (e.g., for drug or materials design), chemistry is the science of transformations in chemical space. In contrast, virtually all ML models for organic molecules to date are trained on reference data derived from the chemical universe database of Reymond and coworkers, which enumerates potentially stable, drug-like molecules^41–43^. Almost by construction, these models therefore cannot describe elementary reactions such as the ones shown in Fig. 1, which typically involve radical or charged intermediates. In our view, the application of ML to areas like catalysis and combustion requires a shift of focus from stable molecules to radicals (i.e., the nodes in Fig. 1) and to reactions (the edges). The goal of this paper is therefore to begin the development of ML models for the exploration of reaction space, as opposed to compound space.

Specifically, we introduce a new DFT database of closed- and open-shell molecules that covers an extensive network of chemical reactions. We then develop ML models to predict atomization and REs. Finally, the models are used to explore the reaction network of methane combustion and identify the most relevant reaction steps and fragments out of a large initial database.

## Results

### Data and kernels

**Fig. 2:**
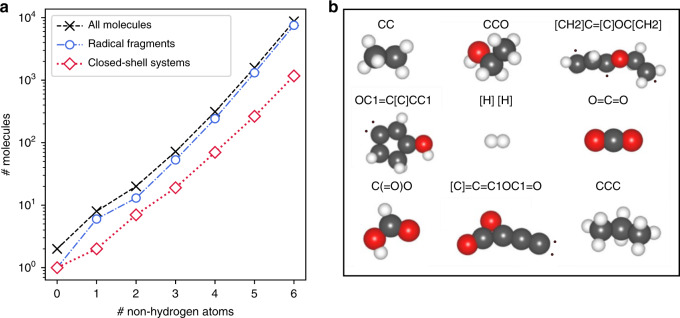
The Rad-6 database. **a** Number of molecules in the database, according to their number of non-hydrogen atoms. **b** Structures of representative molecules in the database. Dots indicate radicals and respective SMILES strings are listed.

To train reactive ML models, a reference database of both open and closed-shell systems must be established. A large set of such structures was enumerated using a graph-based approach^22^, and the ground-state geometry and energy of each system was determined with DFT calculations using the hybrid PBE0 functional with Tkatchenko-Scheffler dispersion corrections^44–46^. The resulting Rad-6 reference database comprises 10,712 molecules containing carbon, oxygen and hydrogen, the largest of which consists of six non-hydrogen atoms. As illustrated in Fig. 2, this dataset is rich in unconventional structural motifs, such as poly-radicals. As is commonly observed, the space of possible compounds scales exponentially with the system size (see Fig. 2, left). This figure also reveals that radical fragments in fact dominate the database, as they are combinatorically much more frequent (by an order of magnitude) than closed-shell systems. Notably, this dominance of open-shell systems prevails, although more than half of the originally enumerated radicals decomposed or rearranged upon geometry optimization. Importantly, these unstable cases were not included in the database. This choice was made because the definition of a chemical reaction requires the specification of the molecular topologies of educts and products (and how they are transformed). The full Rad-6 database is provided in the supporting information to this article.

Two central quantities that are needed to fully understand the overall kinetics of a reaction network are the RE (*E*_reac_, RE) and the activation energy (barrier) for each reaction. Indeed, REs provide the most important features of the reaction network and can in some cases even be used to predict activation energies via the Brønsted-Evans-Polanyi relation^47–49^. Furthermore, while the activation energy is a property of each individual reaction (the edges in a graph), the RE can be computed from molecular atomization energies (*E*_at_, AE), i.e. information from pairs of nodes in a graph, meaning that much fewer calculations are required to predict the REs in a large reaction network. Specifically, to predict 1000 REs for 20 molecules, one only needs 20 ground-state geometries. In contrast, predicting the corresponding activation energies would require 1000 additional transition state (TS) geometries. Not only are there more TS geometries, but these are also much harder to obtain, both in terms of computational effort and in terms of the human intervention needed for successful transition state searches. This makes predicting REs the logical first step in the ML-driven exploration of reaction networks.

Specifically, for a reaction of the type:

1 $$A\,\longrightarrow\, B+C,$$

the REs can be computed from molecular atomization energies via:

2 $${E}_{{\rm{reac}}}={E}_{{\rm{at}}}^{B}+{E}_{{\rm{at}}}^{C}-{E}_{{\rm{at}}}^{A},$$

where we define the AE without loss of generality as the total energy of the molecule minus total energies of the isolated neutral atoms.

Learning atomization energies across chemical compound space is a well-established practice in the ML literature. In a first approach, we can therefore apply such compound space models for predicting REs, as long as they are trained on a reactive database like Rad-6. Herein, we use Kernel Ridge Regression (KRR) with the SOAP^50^ representation, as a state-of-the-art ML method (see SI for details). In brief, KRR uses a kernel function *k*(*x*_*i*_, *x*_*j*_), to measure the similarity between representations *x*_*i*_ and *x*_*j*_. The herein used SOAP representation is one of a class of atom-density projections that have been found to yield highly accurate molecular ML models^51,52^. With this type of model, the AE of an unknown molecule can be predicted according to its similarity with known molecules in a training set. Since the AE is a molecular property and SOAP is an atomic representation, an additional step is required for evaluating the similarity of molecules. This can, for example, be achieved with the average kernel^37^:

3 $${K}_{{\rm{int}}}(A,B)=\mathop{\sum }\limits_{a\in A,b\in B}\frac{1}{{N}_{A}\ {N}_{B}}k({x}_{a},{x}_{b}),$$

where *N*_*A*_ and *N*_*B*_ are the numbers of atoms *a* and *b* in molecules *A* and *B*, respectively, and *x*_*a*_ is the SOAP representation of the chemical environment of atom *a*. The lower-case *k* is used to differentiate the atomic from the molecular kernel function *K*. Alternatively, one can also use the sum kernel:

4 $${K}_{{\rm{ext}}}(A,B)=\mathop{\sum }\limits_{a\in A,b\in B}k({x}_{a},{x}_{b}).$$

Both average and sum kernels have been successfully used in ML models of the AE, but there is a crucial difference in their properties^34,36^: Specifically, the average kernel disregards size differences between molecules. It provides a measure for how similar the atoms in molecule *A* are to the ones in molecule *B*, on average. Meanwhile, the non-normalized sum kernel is sensitive to size differences. Consequently, models using the average kernel should be used to predict intensive quantities, and models using the sum kernel should predict extensive properties^53^. Herein, all models using the average kernel are therefore trained on the atomization energy per atom (AE/*N*, an intensive quantity). The predicted AE/*N* is afterwards simply multiplied with the number of atoms *N* to recover the AE. Meanwhile, the sum kernel can directly be trained on (and predict) the AE^53^. In the following we will refer to Eq. (3) as the intensive kernel (*K*_int_) and to Eq. (4) as the extensive kernel (*K*_ext_). As an aside, it should be noted that using such linear combination kernels is equivalent to the partitioning of the total energy inherent, for instance, to Gaussian Approximation Potentials^29,36^.

To train ML models, the Rad-6 database is split into training, validation (for hyperparameter optimization) and test sets. To obtain representative training sets, we use the farthest point sampling (FPS) method^36^. In FPS, data-points are sequentially selected to maximize the distance between a new data-point (a molecule *A*) and all previously selected points (molecules *B* already in the training set). In the present context, this means new molecules added to the training set should be as dissimilar as possible to all previously selected molecules. The distance between molecules is measured using the previously introduced kernels, according to:

5 $$D(A,B)=\sqrt{K(A,A)+K(B,B)-2K(A,B)}.$$

Because *D*(*A*, *B*) depends on the kernel, we obtain different training sets for the intensive and extensive kernels. Most importantly, while we normalize *K*_int_ so that *K*_int_(*A*, *A*) = *K*_int_(*B*, *B*) = 1, *K*_ext_ is not normalized. Consequently, ${K}_{{\rm{ext}}}(A,A) \sim {N}_{A}^{2}$ and ${K}_{{\rm{ext}}}(B,B) \sim {N}_{B}^{2}$. This means that the distance *D*_ext_(*A*, *B*) evaluated with the extensive kernels tends to be greater between large systems than the distance between small systems. Accordingly, mostly large molecules are selected during the early iterations of FPS with *D*_ext_, whereas the intensive distance *D*_int_ maximizes the average chemical diversity in the training set irrespective of size. It should be noted that a FPS selection based on maximally diverse atomic environments rather than molecules (e.g. using a softmax criterion^54^) would also be possible. This may be a better choice for datasets with large molecules.

**Fig. 3:**
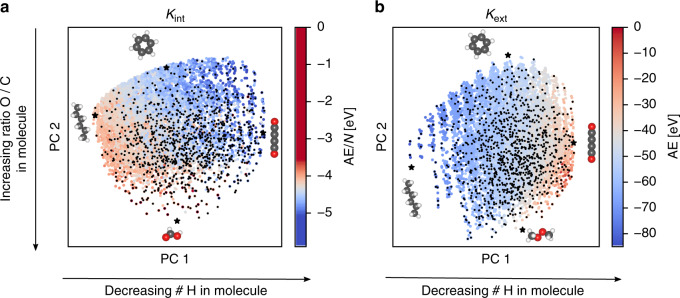
Visualizing Rad-6 with Kernel Principal Component Analysis (kPCA). **a** kPCA based on an intensive kernel. **b** kPCA based on an extensive kernel. Points are colored according to the DFT atomization energy per atom in (**a**) and total atomization energy in (**b**). The arrows provide a qualitative interpretation of the principal component (PC) axes and small black dots indicate the FPS-selected training configurations for a ML model with 1000 training molecules and using the corresponding distance criterion (*D*_int_ (**a**), *D*_ext_ (**b**)), see text.

Beyond their use in regression methods like KRR, kernels can also be used for dimensionality reduction and visualization of large data sets with the kernel principal component analysis (kPCA) method^55,56^. In Fig. 3, kPCA plots of the Rad-6 chemical compound space for the intensive and extensive kernels are shown. Here, the two principal components mainly reflect the degree of saturation (the number of hydrogen atoms) and the oxygen/carbon ratio. The main difference in both projections is that the extensive kernel additionally displays a size-dependence, with small molecules (up to 4 heavy atoms) concentrated in the bottom right corner (see SI for more details).

Superposed on the projected landscapes, Fig. 3 shows the color-coded variation of the DFT computed AEs. A clear trend from more negative values in the top right to less negative values in the bottom left can be discerned for *K*_int_. This correlation of AE/*N* with the degree of saturation results simply because highly saturated molecules contain only single bonds, while unsaturated molecules contain double and triple bonds. The gradual variation of both AE and AE/*N* also provides an intuitive understanding of why kernel models work for predicting molecular energies: Molecules that are close in the kPCA plot (i.e., considered to be similar by the kernel) also have a similar AE. Finally, Fig. 3 also illustrates the distribution of the FPS-selected training points, which evenly cover the compound space, but also span most of the more isolated points at the bottom of the figure.

### Machine learning in compound space

**Fig. 4:**
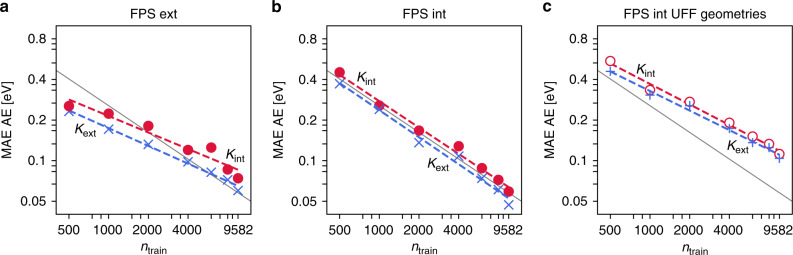
Learning curves for atomization energies (AE). **a** Mean absolute error (MAE) of AE predictions on the test set, as a function of the number of training molecules *n*_train_. The training sets were constructed using FPS with the extensive (**a**) and intensive kernels (**b**) (see text). **c** AE learning curves using molecular geometries obtained with the universal forcefield (UFF). The gray line represents a learning rate of ${n}_{{\rm{train}}}^{0.65}$ and serves as a guide to the eye in all three panels.

In Fig. 4, the learning curves for AE predictions with the extensive and intensive kernels and using both *D*_ext_-based and *D*_int_-based FPS sets are shown, i.e., we also combine extensive kernel learning with intensive training sets and vice versa. It can be seen that with the largest training sets, all four models are able to predict atomization energies for these systems with mean absolute errors (MAEs) well below 0.1 eV. In all cases, the log-log plots display the expected linear relationship (i.e., the learning curve can be fitted as a power law), indicating that even higher accuracy could be achieved with more data. To put this performance into perspective, it should be noted that our baseline method (dispersion-corrected hybrid DFT) itself has an average accuracy of ca. 4–5  kcal mol^−1^ (0.2 eV) for REs and barriers^57,58^.

Additional ML models were trained on randomly sampled training sets, to provide a baseline for the FPS schemes. The corresponding AE learning curves are comparable to the extensive FPS (see SI). As has previously been noted, random sampling is actually advantageous for very small training sets, but the learning rate is lower than for both FPS schemes translating into inferior performance for larger training sets^36^.

Following common practice, all errors are shown for the total AE, even for the intensive models. Clearly, this is not a completely fair comparison, as the intensive models are trained to minimize the AE/*N* and not the total AE error. This explains the seemingly counter-intuitive fact that the extensive model performs better even on the intensive FPS training set. It has been suggested in the context of electronic structure methods that AE/*N* may generally be a more appropriate target for fitting and benchmarking^58,59^. Specifically, fitting on the total AE will selectively favor large systems over small ones, as they offer a larger potential for improvement in the loss function. This also carries over to the FPS selection, as extensive selection will initially focus on larger molecules which are deemed to be more dissimilar than smaller ones. We will see later that this has significant consequences for reaction networks and REs. Nevertheless, based on the data in Fig. 4 one would deduce a slight superiority of the extensive kernel.

Fully optimized DFT geometries will unfortunately not be available for ML training and prediction in a realistic application. If they were, the DFT energy would be known and the ML prediction would be redundant^60^. We therefore also used simple forcefield geometries (based on the universal forcefield, UFF)^61^ for training and prediction, still using the ground-state energies of relaxed DFT geometries as the target property. As shown in Fig. 4c and detailed in the SI, all trends discussed for the DFT geometries are unchanged, but the MAEs are somewhat higher, roughly by a factor of two. Such inferior performance of ML models using approximate geometries has also been observed for closed-shell data sets like QM9, but it is more pronounced here^36^. This reflects the fact that general forcefields like UFF are not designed for the description of radicals, which make up a large part of Rad-6. In this context, semi-empirical electronic structure methods might offer an alternative low-cost method for more reliable geometries^62,63^. Note however that such methods will invariably afford some amount of rearrangement and decomposition upon geometry optimization, which would introduce a mismatch between the structure used to build the SOAP representation and the structure for which the target energies are computed. This could in principle be mitigated by using constrained relaxations, but defining universal geometrical constraints in a high-throughput setting is not trivial.

It has also been shown that predictions from approximate geometries can be improved by using a measure of the quality of the training geometries to adjust the model regularization for each training sample^36^. As shown in the SI, this is not successful for Rad-6. Again, we attribute this to the overall poor and inconsistent quality of the UFF geometries for open-shell systems, highlighting another challenge when moving towards ML approaches for reaction space.

**Fig. 5:**
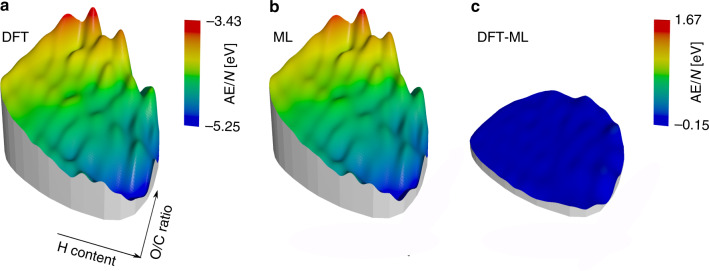
Illustration of the Rad-6 chemical space as an interpolated height profile. **a** kPCA as in Fig. 3 showing the DFT reference intensive atomization energies AE/*N* (in eV). **b** Prediction from the ML model using an intensive kernel and a small intensively selected training set of only 1000 molecules with UFF geometries. **c** Respective differences (DFT-ML). Here, the range of the colorbar is shifted but the scale is the same.

Nonetheless, even UFF-based models with fairly small training sets already provide a reasonable estimate of the AEs across chemical compound space. This is illustrated in Fig. 5, where an interpolated AE/*N* surface for an ML model trained on 1000 UFF structures is compared to the DFT reference values. The plots are visually almost indistinguishable. This serves to emphasize that even a ML model trained on 10% of the database already provides an adequate representation of its overall thermochemistry. Recall that the core task for the development of rationally reduced reaction networks is not an excessive accuracy of this thermochemistry as typically targeted in existing ML work for compound space. Instead, the overall topology needs to be appropriately represented to a degree that enables the selection or dismissal of reactions when building sub-graphs.

### Machine learning in reaction space

With the ML-predicted AEs, one can readily calculate REs using Eq. (2), in strict analogy to how they are computed with first-principles methods. In this case, errors in the predicted AEs will propagate to the predicted REs. Under the most basic assumptions (i.e., an uncorrelated, constant uncertainty *σ*_*AE*_ for every AE prediction), one would expect the uncertainty in the RE prediction for a reaction *A* ⟶ *B* + *C* to be $\sqrt{3}{\sigma }_{\mathrm{AE}}$. While this is a very rough estimate, it indicates that we would generally expect the error on REs to correlate with the AE error, and that the former should be larger than the latter.

**Fig. 6:**
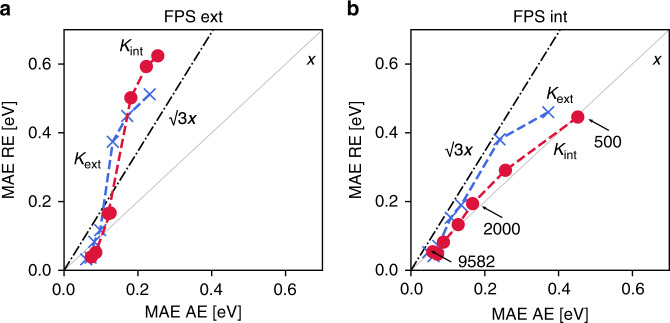
Correlation of mean absolute errors (MAE) for AE and RE prediction. **a** Correlation plot for the extensive FPS training set using the extensive and intensive kernels and DFT geometries. **b** Correlation plot for the intensive FPS training set using the extensive and intensive kernels and DFT geometries. Multiple points for each model represent the different training set sizes shown in Fig. 4 (indicated in (**b**)), with smaller AE errors corresponding to larger training sets.

To test these expectations, a reaction network containing 32,515 bond-breaking reactions, Rad-6-RE, was generated using the Rad-6 molecules (see SI for details and the full dataset). In Fig. 6, we show the relation between the performance of different ML models for AE and RE predictions, using both FPS training set selections (multiple points for each method correspond to the different training set sizes shown in Fig. 4). These plots reveal several interesting trends. As expected, the RE error correlates with the AE error. However, there are significant differences both with respect to the FPS selection and the kernels. Most notably, all models display unexpectedly large errors for the smaller (*N* ≤ 2000) extensive training sets. In contrast, the models trained on the intensive FPS display RE errors that are much closer to the corresponding AE errors. Strikingly, the combination of intensive kernel learning and intensive training set selection leads to RE errors that are almost identical to the corresponding AE errors across all training set sizes.

These observations can be understood in light of the fact that not all molecules are equally weighted in a reaction network. As can be seen in Fig. 1, some molecules are central hubs in the network (dark green), whereas others lie on the periphery and only contribute to few reactions (white)^11,19^. The existence of such hubs, which correspond to molecules with dramatically higher importance, is a fundamental difference between reaction space and the homogeneously weighted chemical compound space. In Rad-6-RE, the most important such hubs are small molecules that correspond to functional groups (OH, CH_3_, etc.) and the isolated atoms C, H and O. As mentioned previously, the extensive kernel distance *D*_ext_ will consider all smaller molecules to be more similar in terms of their kernel distance (Eq. (5)), because the terms *K*_ext_(*A*, *A*) and *K*_ext_(*B*, *B*) scale with the number of atoms. Small molecules are therefore selected later in an extensive FPS selection, and are consequently absent from the smaller training sets. This can lead to relatively large errors on important hub molecules, which will consequently have an out-sized impact on the RE error.

In other words, the large discrepancy between RE and AE for small extensive training sets is because small molecules are less likely to be included. This notion is further reinforced by considering the performance of the models based on random sampling. While the AE predictions of these models are of comparable accuracy with the FPS models (in particular for the smaller training sets), the performance for RE prediction is very poor, with MAEs above 1 eV for small training sets (see SI). Even when the extensive kernel is trained on intensive sets, smaller molecules still offer less potential for improving the loss function and thus lead to a poorer performance for REs.

In complete contrast to the situation in compound space, an intensive kernel with an intensively selected training set is therefore a better choice for ML models in reaction space. This indicates that some of the experience gathered hitherto for ML in chemical compound space (like the significant work on the QM9 database)^34–36^ will not necessarily carry over to reaction spaces. Realizing the particular relevance of hub molecules, a straightforward adaptation could for instance simply be to inversely scale the extensive distance used in the FPS selection by the degree of the node in the reaction network, i.e., by the number of reactions in which the molecule is involved (see Fig. 1). Similarly, the least-squares problem for an extensive kernel could be adjusted by weighting the molecules according to their inverse size. With this work, we hope to initiate such dedicated methodological development for reaction spaces and will pursue corresponding research in the future.

It should also be noted that the special topology of reaction networks makes model evaluation for REs in a strict statistical learning framework difficult. The reaction network Rad-6-RE contains most of the Rad-6 molecules. Computing the REs for this network is therefore not a pure prediction, as some molecules in each reaction may be in the training set. In principle, it would be desirable to evaluate the performance on a separate reaction network that contains no training molecules at all. However, this can only be achieved in two ways: Either the test network contains no small molecules like CO and OH, or these molecules are excluded from the training set. The former option leads to a very unnatural reaction network, that misses the most frequent classes of bond-breaking events. Meanwhile, the latter option leads to a very poor training set, and thus an overly pessimistic estimate of model performance.

We therefore decided not to follow this strict separation of training and prediction for the RE MAEs shown in Fig. 6. This also explains why the RE error is in some cases actually lower than the AE error, contrary to expectation: The RE MAE benefits from the fact that the prediction error of all tested models is somewhat lower on the training sets (see SI). Indeed, KRR models can in practice display a negligible error on the training set if the regularization parameter is chosen to be very small, as is advocated by some authors^64^.

### Exploration of reaction networks

Finally, we return to the original motivation of this work, namely the ML-aided exploration of complex reaction networks. To illustrate the use of ML-predicted REs, we consider a closed network of over 21,393 elementary reactions, containing a large variety of bond-breaking, transfer and rearrangement reactions for oxygen, carbon and hydrogen-containing molecules^22^. Note that this network is deliberately not a subset of Rad-6, although there is significant overlap (ca. 80% of the involved molecules are included in Rad-6). This is thus, at least partially, an out-of-sample application. The challenge lies in determining which of the elementary reactions are likely relevant to a chemical process of interest. As an exemplary process we consider the early stages of methane combustion^65–69^.

To validate the proposed ML models for this application, additional DFT calculations were performed on the out-of-sample systems. Unfortunately, these systems mostly decompose or rearrange upon DFT geometry optimization. Note that this does not necessarily mean that they are inherently unstable, however, just that the corresponding local minima were not found when starting from a (inaccurate) UFF geometry. We therefore used DFT single point calculations on UFF geometries here. Overall, a good correlation between DFT and ML-predicted energies is found, with systematically lower ML AEs (see SI). This systematic bias can easily be understood since the ML models predict the DFT energies of relaxed geometries, but the validation energies are for frozen UFF geometries. The latter is by definition larger than the former. This shows that the ML model can be used to estimate relaxed DFT energies even when these are not readily available from DFT calculations.

To qualitatively explore this network, a mean-field microkinetic simulation of the reaction of equal parts CH_4_ and O_2_ was performed, assuming a constant activation barrier for all reactions (see SI for details). Under these assumptions, the reaction dynamics are only driven by the REs and the law of mass-action. While the true activation energies and detailed reaction conditions (initial concentrations, temperature, pressure, etc.) will obviously play a crucial role for the actual mechanism, such a simplified microkinetic simulation provides insight into how thermochemistry and the topology of the reaction network define which intermediates and reaction steps are at all relevant to the process. By observing how the reaction network grows with simulation time, we can furthermore understand how intermediates and reactions sequentially become available, as mass flows through different paths of the network. Only requiring ML-predicted REs as input, such a simulation is therefore a first step towards the envisioned rational reduction of the full network to tractable sub-graphs.

**Fig. 7:**
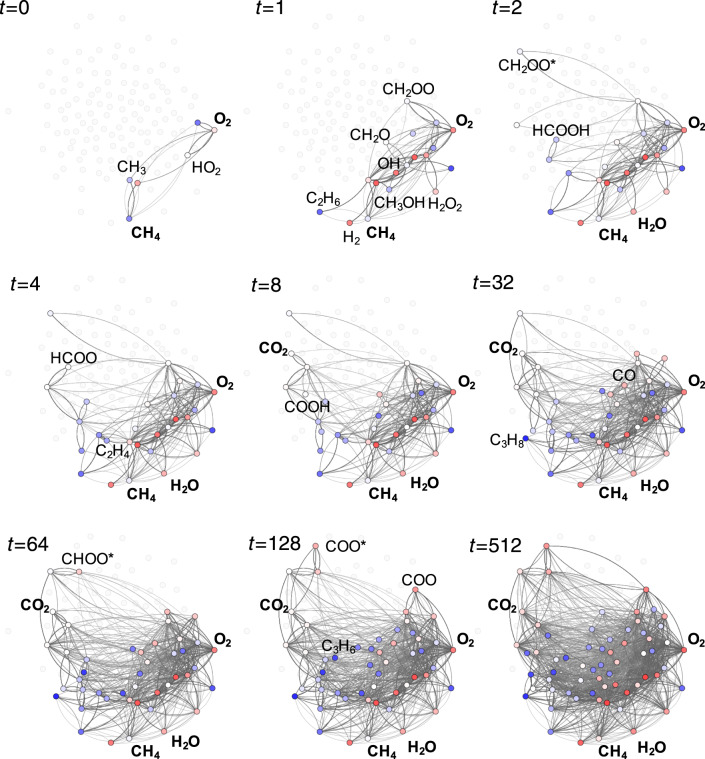
ML-based exploration of a complex reaction network. Each frame shows the reduced reaction network extracted from a microkinetic simulation of methane combustion at different stages in simulation time. The abstract simulation time is shown for each frame in arbitrary units, see text. Educts and products (in bold), as well as important intermediates are highlighted. Nodes are colored according to their absolute atomization energies from low (red) to high (blue). Cyclic compounds are marked with an asterisk, to distinguish them from the corresponding linear compounds.

  Figure 7 summarizes the results obtained based on the intensive kernel ML model trained with an intensively selected FPS set of 9582 UFF structures. Shown are the reduced reaction networks extracted as those parts of the full network that are accessed at increasing simulation times. These reduced networks are highly revealing, as they form a hierarchy of different chemistries relevant to combustion. For example, in line with general expectations^6^, the smallest network contains peroxide chemistry, with the hydrogen transfer from methane to molecular oxygen as the dominant pathway. Subsequently formed CO_*y*_H_*x*_ intermediates also comprise generally anticipated molecules like methanol (CH_3_OH) or formic acid (HCOOH), but also more exotic species like the Criegee intermediate (CH_2_OO). Interestingly, the formation of the main product CO_2_ only appears in larger subgraphs after dimerization reactions have already led to C_2_ intermediates like ethylene (C_2_H_4_) and ethane (C_2_H_6_). Finally, the largest subgraphs shown include already more complex molecules like propane (C_3_H_8_) and propene (C_3_H_6_) and comprise a total of 887 reactions.

It should be emphasized that the networks in Fig. 7 are not intended to represent a definitive mechanism for methane combustion, not least because this mechanism strongly depends on reactions conditions like temperature, pressure and the methane/oxygen ratio^68^. Instead, this analysis provides insight into what intermediates and elementary steps should be considered when constructing reduced reaction networks for mechanistic studies. While assuming constant barriers is clearly a harsh approximation in a microkinetic simulation, we note that predicting activation energies for the full network is not necessary to extract the relevant reduced reaction network for subsequent analysis. In many cases, an elementary reaction can be discarded because of a large thermochemical barrier alone. In other words, if a reaction is found to be irrelevant in a microkinetic simulation with constant barriers, it will not become relevant once activation barriers are included. Of course, activation barriers for the reduced network must still be computed for a quantitative microkinetic simulation, but this is only a small subset of the full network.

Note also that a pure ML approach may miss important domain knowledge. For example, both singlet and triplet spin-states of CH_2_ are relevant in combustion^6,68^. Instead, the graph-based enumeration approach^22^ used to generate Rad-6, generically only considers the lowest-spin state of each molecule (with manually implemented exceptions of triplet O_2_ and the isolated atoms to prevent completely unphysical results). Nonetheless, our pure ML approach finds all intermediates considered in empirical reduced methane combustion mechanism like the skeletal mechanism of Lu et al.^6,70^. On the other hand, the unbiased nature of ML approaches has the benefit of providing unexpected suggestions that would perhaps not be considered otherwise. For example, already our proof-of-concept reduced reaction networks of methane combustion suggest a pathway for CO_2_ formation via the Criegee intermediate (CH_2_OO) and cyclic compounds like dioxirane (CH_2_OO*) that is not generally considered in state-of-the-art empirical networks. In our view, domain knowledge and ML-based exploration should therefore be combined in practice.

Indeed, the generation of reference databases is also to an extent domain specific. The reaction networks considered herein are quite universal and could be applied to atmospheric chemistry, combustion or catalysis. However, these fields have distinct requirements with respect to the first-principles reference data. Clearly, catalysis can only be studied if the effect of the catalyst is accounted for. Meanwhile, thermal contributions to the free-energy will be large and important for a realistic description of combustion, and the role of different spin-states must be considered in both combustion and atmospheric chemistry. Nevertheless, the ML framework presented herein can easily be transferred to accommodate these situations.

**Fig. 8:**
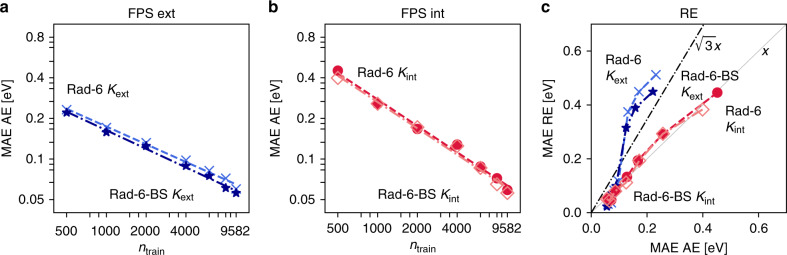
Comparison ML models trained on the Rad-6 and the Rad-6-BS databases. **a** Learning curves for AE predictions of using the extensive kernel with an extensive FPS split and DFT geometries. **b** Same as (**a**) but for the intensive kernel with an intensive FPS split. **c** Correlation plot of MAE RE vs MAE AE for both Rad-6 and Rad-6-BS. Blue lines represent results obtained with the extensive kernel (crosses for Rad-6 and stars for Rad-6-BS) in (**a**) and (**c**). Red circles correspond to the intensive kernel with Rad-6 and orange diamonds to the intensive kernel with Rad-6-RE.

To demonstrate this, a second set of energies for Rad-6 was computed using broken-symmetry (BS) DFT (see SI for details). In BS-DFT, the DFT energy is further minimized by exploiting the breaking of spatial and spin-symmetry in the Kohn-Sham determinant. The resulting determinants consequently do not correspond to a predefined multiplicity but represent the lowest energy solution irrespective of the spin state. Importantly, we find that ML models trained on this data have very similar predictive accuracy to the ones discussed so far (see Fig. 8). This shows that the Rad-6 database can serve as a benchmark for developing and improving ML models in reaction space, much like the popular QM9 set has done for chemical compound space.

## Discussion

In this paper, we have explored the applicability of ML models to chemical reaction networks. In this context, we introduced the Rad-6 database of ca. 10,000 open and closed-shell molecules and an associated reaction network of ca. 30,000 reactions (Rad-6-RE). Established compound space KRR methods were shown to accurately predict atomization energies of the Rad-6 molecules. While the AE prediction accuracy was fairly similar for different choices in training set selection and kernel construction, these choices had a large effect on RE prediction accuracy. In particular, we found the use of an intensive kernel for both FPS-based training set selection and KRR learning to work very well for RE prediction, while models trained on extensive FPS sets displayed unexpectedly large RE errors. This can be rationalized by the special topology of reaction networks, in which certain small molecules constitute important hubs that should be included early on in the training sets.

We note that the extensive and intensive kernels used herein are merely interesting representatives of a wider range of possible models. Fundamentally, the observed differences in performance between the AE and RE prediction reflect that not all concepts established for the ML-based exploration of chemical compound space can be carried over to reaction space. Multiple methodological developments are required to establish reliable protocols, for example with respect to the weighting of molecules in the loss function of the ML model. If the topology of the reaction network of interest is known, these weights could for example be selected according to the connectivity of the molecule in the network (as shown in Fig. 1). Alternatively, weighting by size (or molecular weight) would likely be a useful heuristic to avoid the problems observed for the extensive kernel.

We also presented a proof-of-principle application of a reactive ML model to the exploration of the methane combustion reaction network. Here, a microkinetic simulation based on ML energetics was carried out, revealing relevant pathways and elementary steps in a large reaction network of 21,000 reactions. In our view, there are two ways to proceed from here. On one hand, the relevant subgraph thus extracted from of a much larger reaction network could be studied in depth with first-principles methods. On the other hand, we can envision an ML-driven computational reactor, where this is done in a more integrated fashion. Important steps (as identified by an ML-driven microkinetic simulation) could be studied with DFT and the results used to retrain the ML model. This would lead to an active-learning-type iterative procedure, where the predicted energetics of the reaction network are continuously improved in a targeted fashion, and no subgraph selection is necessary (within the computational constraints of the microkinetic simulation).

## Methods

### Computational details

Reference geometries and energies were obtained using DFT as implemented in FHI-Aims^46,71^. Specifically, the PBE0 functional^72^ was used with tight integration settings and tier-2 numerical atomic orbital basis sets. Dispersion interactions were treated via the pair-wise Tkatchenko-Scheffler van-der-Waals correction^73^. Approximate geometries were obtained with the UFF forcefield^61^.

### Machine-learning models

All reported ML models are based on Kernel Ridge Regression and use the SOAP kernel^37,50^. SOAP representations were computed with the quippy code (https://github.com/libAtoms/QUIP). Kernel matrices and training/test splits were generated with the mltools package (https://github.com/simonwengert/mltools.git). The atomic simulation environment was used throughout to process molecular data^74^.

Full methodological details are provided in the Supplementary information.

## Supplementary information

Supplementary Information

Description of Additional Supplementary Files

Supplementary Data 1

## Data Availability

All datasets used in this paper are available as Supplementary Data 1.

## Code availability

The code used to fit the ML models is available at 10.5281/zenodo.4025972.
