# Supplementary material for: Machine learning in chemical reaction space
Source: Nat Commun. 2020 Oct 30;11:5505. doi: 10.1038/s41467-020-19267-x (PMC7603480; doi:10.1038/s41467-020-19267-x)
Supplement: Supplementary file 1 — Supplementary Information [file 41467_2020_19267_MOESM1_ESM.pdf]

Supporting Information - Machine Learning in Chemical Reaction Space

Sina Stocker,[1] Gábor Csányi,[2] Karsten Reuter,[1,3] and Johannes T. Margraf[1]

[1] *Chair of Theoretical Chemistry and Catalysis Research Center, Technische Universität München, Garching, Germany*

[2] *Engineering Laboratory, University of Cambridge, Cambridge CB2 1PZ, United Kingdom*

[3] *Fritz-Haber-Institut der Max-Planck-Gesellschaft, Berlin, Germany*

(Dated: 25 September 2020)

Supplementary Note 1: Rad-6 Database

The Rad-6 reference database comprises both closed-shell molecules and (poly-)radical fragments containing carbon, oxygen and hydrogen (see below for a detailed description). SMILES strings[1] for structures containing up to 6 non-hydrogen atoms were created using the graph-based approach of Margraf and Reuter[2] and subsequently converted to 3D structures using the RDKit package.[3] Geometries were initially relaxed with the universal forcefield (UFF).[4] Final geometries and energies were obtained using DFT as implemented in FHI-Aims[5,6]. Specifically, the PBE0 functional[7] was used with tight integration settings and tier-2 numerical atomic orbital basis sets. Dispersion interactions were treated via the pair-wise Tkatchenko-Scheffler van-der-Waals correction.[8] The final reported geometries are converged to a maximum residual force component of $10\,\text{meV}\,\text{Å}^{-1}$ per atom.

As ML models do not explicitly consider electronic structure, special considerations with respect to spin states are required. In Rad-6, all DFT calculations were initialized with low-spin densities (singlet multiplicity for even number of electrons, doublet for odd number of electrons), constructed according to the location of radical electrons in the SMILES string. Exceptions were made for the carbon and oxygen atoms as well as for the oxygen molecule, which were treated as triplets. This was to ensure correct atomization energies and reasonable energetics for oxidation reactions with O_2. All open-shell systems were treated with collinear spin-polarization. These choices are arbitrary, but inconsequential to the conclusions of this study. A rigorous treatment of spin in a ML context is in principle possible, but this would require fitting a separate model for each spin-state.

A second important issue relates to the geometries used for ML. Many of the initially constructed poly-radicals decompose during the DFT relaxation or simply do not converge. This is problematic for two reasons: Firstly, the definition of chemical reactions presupposes a certain molecular topology (i.e. how atoms are connected). Secondly, in this case the UFF geometry (with fixed topology) describes a different molecule than the DFT one. In a realistic setting, the DFT geometries will not be available for ML predictions. If they were, the DFT energy would also be known and the ML prediction would be redundant.[9,10] In the main manuscript, both UFF and DFT geometries are used for training and prediction, but the energies of relaxed DFT geometries are always the target property (see Supplementary Figure 1).

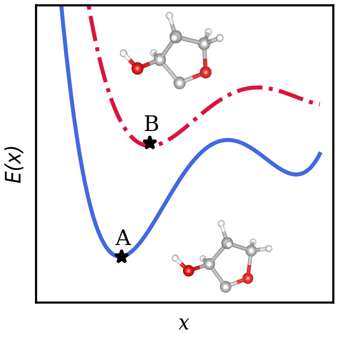

Supplementary Figure 1. Schematic representation of a DFT (blue, solid line) and forcefield (red, dashed line) potential energy surface. As it is mentioned in the text, two different types of ML models are used in this work. (1) The ML models predict the energies of relaxed DFT geometries (point A) and the corresponding DFT geometries (point A). (2) The ML models predict the energies of relaxed DFT geometries (point A) based on structures relaxed with a forcefield (point B).

In order to allow for a clear definition of reactions in terms of bond-breaking and formation, only those systems where UFF and DFT geometries describe the same molecular topology are included in the database. This leads to a drastic reduction from the initial set of over 27,000 systems to 10,712 structures in the final Rad-6 database. A positive side effect of this is that the structures in Rad-6 can be expected to be reasonably stable, since they represent local minima on the DFT calculated potential energy surface.

Rad-6-BS database: To investigate the stability of the proposed ML approach with respect to changes in the data (in particular regarding the spin-state), a second set of single-point energies was calculated for the Rad-6 database, using broken-symmetry DFT. Here, calculations were performed with the revPBE functional and def2-TZVP basis-set using Orca.[11,12] To enable symmetry breaking even for nominally closed-shell systems, the beta-spin orbitals in the initial guesses were perturbed by randomly mixing an occupied and unoccupied orbital. After convergence, four additional calculations were performed for each system, reusing the converged wavefunctions from previous runs and further perturbing the orbitals. This procedure was used to avoid SCF convergence into local minima or saddle points.[13]

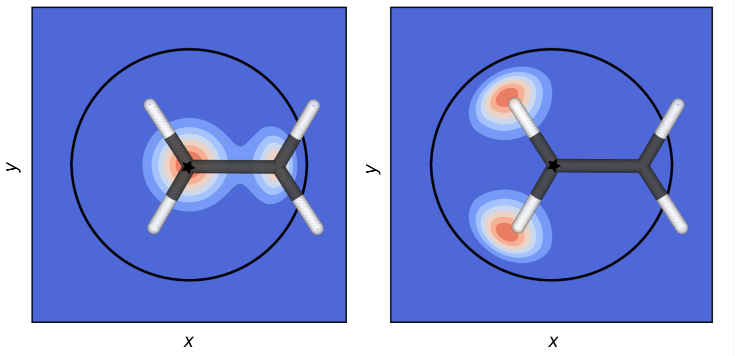

Supplementary Figure 2. The smooth overlap of atomic positions (SOAP) kernel uses a three-dimensional neighborhood density function $\rho_a^Z(\mathbf{r})$ of broadened atomic positions within a cutoff. As mentioned in the text, for every species a separate density is constructed. Planar cuts through $\rho_a^{\mathrm{C}}(\mathbf{r})$ (left) and $\rho_a^{\mathrm{H}}(\mathbf{r})$ (right) around a cutoff centered carbon atom (star) in ethylene are shown. The black circle represents the radial cutoff distance.

Supplementary Note 2: Theory and Computational Methods

Smooth overlap of atomic positions (SOAP): SOAP is a local kernel that measures the similarity of atomic environments.[10] It was found to be highly successful in molecular and solid-state applications.[10,14–16] Below, a brief overview of the concept is given, more details can be found in the literature.[17,18]

SOAP is based on the neighborhood density function $\rho_a(\mathbf{r})$ around a reference atom a:

$$\rho_a(\mathbf{r}) = \sum_{i \in \chi_a} \exp\left(-\frac{(\mathbf{r} - r_{ai})^2}{2\sigma_{\mathrm{at}}^2}\right) \times f_{cut}(\mathbf{r}) \tag{1}$$

where the sum runs over all neighboring atoms i (within a cutoff radius, the atomic environment χ) and $f_{cut}(\mathbf{r})$ is a damping function ensuring that the density smoothly approaches zero at the cutoff. Each atom (including the reference atom) within the cutoff is broadened with a Gaussian of width σ_{at}, leading to a smooth, local representation of the atomic environment.

The atom centred neighborhood density in Supplementary Eq. 1 complies with a system containing only one type of atomic species. For systems with different types of elements, like molecules, the density is individually constructed for every atomic species (Z) within

the atomic environment χ of atom a (see Supplementary Figure 2):

$$\rho_a^Z(\mathbf{r}) = \sum_{i \in \chi_a^Z} \exp\left(-\frac{(\mathbf{r} - r_{ai})^2}{2\sigma_{\text{at}}^2}\right) \times f_{cut}(\mathbf{r}). \tag{2}$$

The similarity between two such environments can be measured via a rotationally averaged overlap integral:

$$\tilde{k}(\chi_a, \chi_b) = \int d\hat{R} \left| \int \sum_Z \rho_a^Z(\mathbf{r}) \rho_b^Z(\hat{R}\mathbf{r}) d\mathbf{r} \right|^2 \tag{3}$$

where the outer integral is over all rotations $\hat{R}$, so that $\tilde{k}(\chi_a, \chi_b)$ is invariant to rotations or permutations of atoms. The power of two in the inner integral ensures that the kernel retains angular information about the neighborhood density.

Importantly, this integral can be solved analytically, if the neighborhood density is expanded in an atom-centered basis of orthogonal radial basis functions $g_n(|\mathbf{r}|)$ and spherical harmonics Y_{lm}:

$$\rho_{\chi_a}^Z(\mathbf{r}) = \sum_{nlm} c_{nlm}^Z g_n(|\mathbf{r}|) Y_{lm}(\mathbf{r}). \tag{4}$$

The coefficients c_{nlm}^Z are then transformed into the so-called power spectrum for individual species:

$$\mathbf{p}_{nn'l}^{Z_1 Z_2} = \pi \sqrt{\frac{8}{2l+1}} \sum_m (c_{nlm}^{Z_1})^\dagger c_{n'lm}^{Z_2} \tag{5}$$

which we truncate at n $\leq$ 8 and l $\leq$ 8.

The kernel from Supplementary Eq. 3 can now be computed as a simple dot product of the 'partial' power spectra:

$$\tilde{k}(\chi_a, \chi_b) = \sum_{Z_1 Z_2} \mathbf{p}_{Z_1 Z_2}(\chi_a) \mathbf{p}_{Z_1 Z_2}(\chi_b) \tag{6}$$

To obtain the final SOAP kernel, this function is normalized and squared so that:

$$k(\chi_a, \chi_b) = \left(\frac{\tilde{k}(\chi_a, \chi_b)}{\sqrt{\tilde{k}(\chi_a, \chi_a)\tilde{k}(\chi_b, \chi_b)}}\right)^2. \tag{7}$$

The atomic kernels are the basis to build global kernels for structure matching (e.g. molecules) instead of local environments. Here, we use the average (Eq. 4) and sum kernel

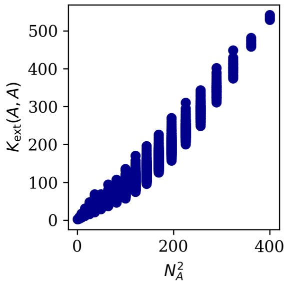

Supplementary Figure 3. Kernel diagonal elements against the number of atoms in a molecule squared. The plot shows that $K_{\mathrm{ext}}(A, A) \sim N_A^2$.

(Eq. 5) described in the main text. It is worth stressing that the average kernel, an intensive kernel has to be normalized:

$$K(A, B) = \frac{\bar{K}(A, B)}{\sqrt{\bar{K}(A, A)\bar{K}(B, B)}}, \tag{8}$$

while the sum kernel should not, i.e. $K(A, B) = K^{\Sigma}(A, B)$. Consequently, the magnitude of the diagonal elements of the sum kernel matrix scales with the square of the number of atoms in the molecule (see Supplementary Figure 3)

In this work, we use the `quippy` code[19] and the `mltools` package[20] to compute SOAP kernels. In order to have flexibility in the description of short and mid-range contributions, a kind of 'multiscale' (ms) SOAP is used. Specifically, two global SOAP kernels with cutoff values of 2 Å (K$_2$) and 4 Å (K$_4$) are applied simultaneously. We use σ_{at} of 0.3 Å for K$_2$ and σ_{at} of 0.6 Å for K$_4$. We combine short and mid-range contributions for the average kernel as the average of the normalized kernels K$_2$ and K$_4$, i.e. $K_{\mathrm{int}}^{\mathrm{ms}} = \frac{K_{2,\mathrm{int}} + K_{4,\mathrm{int}}}{2}$. For the sum kernel we simply sum up the individual sum kernels $K_{\mathrm{ext}}^{\mathrm{ms}} = K_{2,\mathrm{ext}} + K_{4,\mathrm{ext}}$.[21]

Kernel ridge regression: Kernel ridge regression (KRR) is a supervised machine learning technique to obtain function values for given input configurations x_i. In this section we give a short overview about this technique, however for a detailed description and mathematical derivations the reader is referred to literature.[22]

The function can be expressed as linear combinations of kernel functions $(K(x_i, x))$:

$$f(x) = \sum_{i}^{N} \alpha_i K(x_i, x), \tag{9}$$

while the kernel functions act as similarity measures between different input configurations x and x_i with target properties y and y_i. The x_i are feature vectors of training data

points and α_i are regression weights.

KRR provides a closed-form solution for the optimal set of weights α. This can be obtained by minimizing the loss-function l (of a regularized least-squares problem):

$$l = \sum_{j}^{N} \left(\sum_{i}^{N} \alpha_i K(x_i, x_j) - y_j \right)^2 + \sigma \alpha^T \mathbf{K} \alpha \tag{10}$$

The solution of this problem is than given in matrix vector notation:

$$\alpha = (\mathbf{K} + \sigma \mathbf{I})^{-1} \mathbf{y}, \tag{11}$$

where $\mathbf{K}$ is the kernel matrix of the training set (with $K_{ij} = K(x_i, x_j)$), σ is the regularization parameter and $\mathbf{I}$ is the identity matrix. σ is a hyperparameter that has to be determined empirically (see Supplementary Note 3). It represents the noise level of the reference data and is used to control over- and underfitting.

In our work we applied mean-correction to the observables in the fit with the intensive kernel while we did not for the extensive kernel.

Kernel principal component analysis (kPCA): Principal component analysis is a tool for projecting high dimensional data into a lower dimensional space and therefore enables the visualization of that specific data. In PCA, data is transformed into a new coordinate system such that the new coordinate axes point into the direction of largest variance (first coordinate into the direction of largest variance, so-called PC 1, second coordinate into the direction of second largest variance and orthogonal to PC 1, so-called PC 2, ...). Kernel PCA is an extension to PCA and makes the dimensionality reduction of non-linear data possible.[23]

To this end, the kernel matrix is constructed analogous to KRR and then 'centralized':

$$\hat{\mathbf{K}} = \mathbf{K} - \mathbf{1}_{\mathrm{N}} \mathbf{K} - \mathbf{K} \mathbf{1}_{\mathrm{N}} + \mathbf{1}_{\mathrm{N}} \mathbf{K} \mathbf{1}_{\mathrm{N}}, \tag{12}$$

where $\mathbf{1}_{\mathrm{N}}$ is a matrix with the same dimensions as the kernel matrix, in which every element is identically $1/N$ (with the number of data points N). For $\hat{\mathbf{K}}$, the eigenvalue problem has to be solved,

$$\hat{\mathbf{K}} \mathbf{v}_i = \lambda_i \mathbf{v}_i, \tag{13}$$

where $\mathbf{v}_i$ is the ith eigenvector and λ_i the respective eigenvalue. The data can be projected into the new space via:

$$\mathbf{PC}_i = \mathbf{K} \mathbf{v}_i. \tag{14}$$

Supplementary Note 3: Training Set Selection, Hyperparameter Search and Learning Curves

Training set selection: We divide the Rad-6 database into a training, validation (100 structures) and test set (1030 structures). The farthest point sampling (FPS) technique is used to select representative and diverse training configurations. The FPS algorithm starts with an arbitrary data point and sequentially adds new structures so that the distance between the newest structure and all previously selected ones is maximized.[10,18,21] This requires a distance matrix that is constructed using the kernel, according to:

$$D(A,B) = \sqrt{(K(A,A) + K(B,B) - 2K(A,B))} \tag{15}$$

A sequence is generated for the complete Rad-6 database. The last 1030 structures went into test set and 100 structures before the last 1030 into the validation set. Since the distance matrix is a function of the kernel, we obtain different training, validation and test sets for the average and sum kernel. In this work the FPS is done with K_{int} and K_{ext} using UFF geometries and started with the H-atom, respectively.

Hyperparameter search: Our ML models contain several hyperparameter in the SOAP kernel and one hyperparameter in kernel ridge regression. In this work we do not focus on the optimization of the hyperparameter in the SOAP kernel, but optimize the σ hyperparameter in KRR. This is done by evaluating the RMSE of the validation set in a grid search.[9] The results for all kernels and FPS splits are shown in Supplementary Figures 4-7.

Including also the hyperparameter optimization for the SOAP kernels could lead to even smaller errors on the predictions.

Learning curves: Learning curves of AE and RE for the respective kernels and FPS splits are shown in Supplementary Figures 8-11. These plots show the MAE and RMSE for training, validation and test set (two left subplots) as well as for the reaction network Rad-6-RE (two right subplots).

Supplementary Note 4: Learning AE with UFF Geometries

Supplementary Figure 12 displays the results for the predictions of atomization energies using DFT geometries (Fig. 4 main text) as well as the MAEs for AE using UFF geometries. As mentioned in the main text, using UFF instead of DFT geometries leads to the same

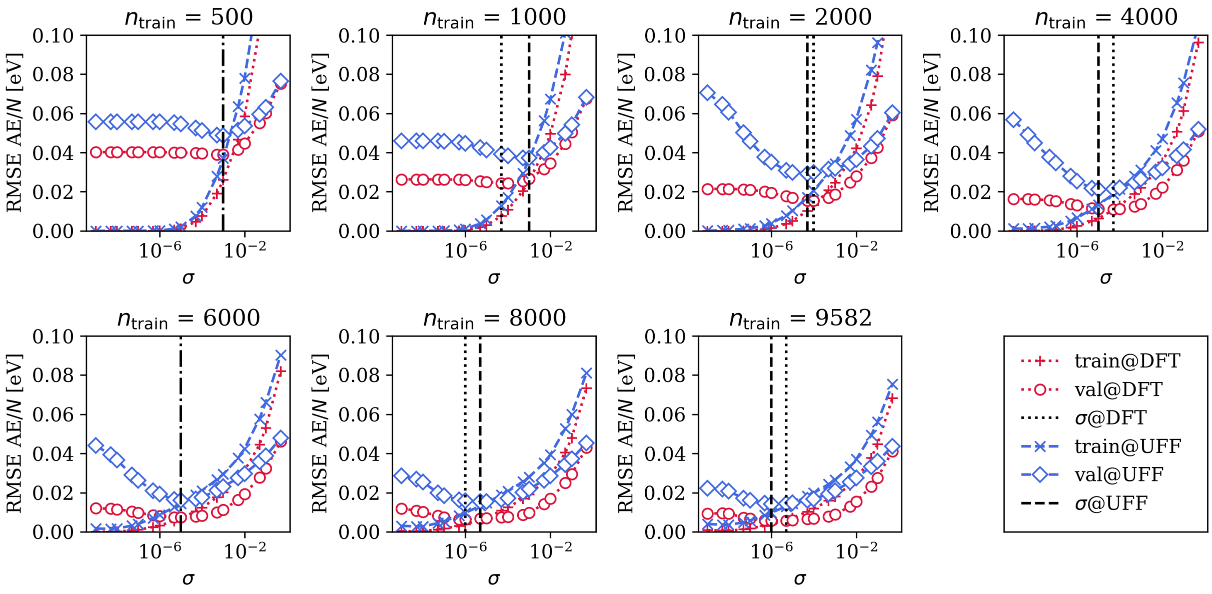

Supplementary Figure 4. Hyperparameter search for K_{int} FPS int.

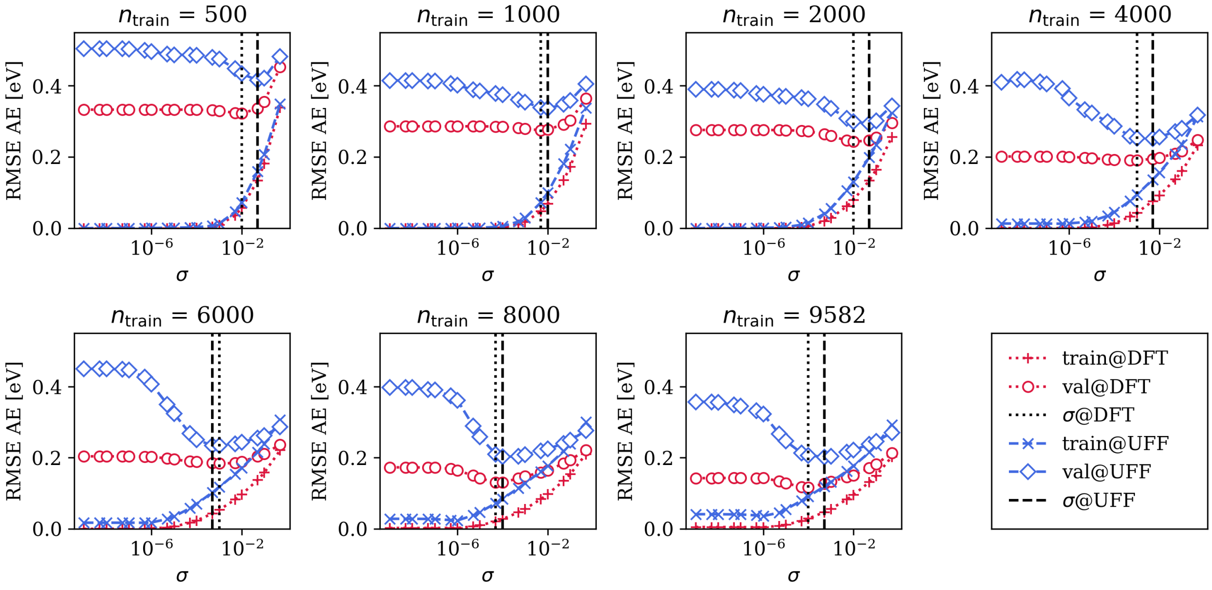

Supplementary Figure 5. Hyperparameter search for K_{ext} FPS ext.

trends in learning curves for the different kernels and FPS splits. However, it results in higher errors on the predictions since the ML model has to additionally learn the differences between the geometries for the different levels of theory (see. Supplementary Figure 1).

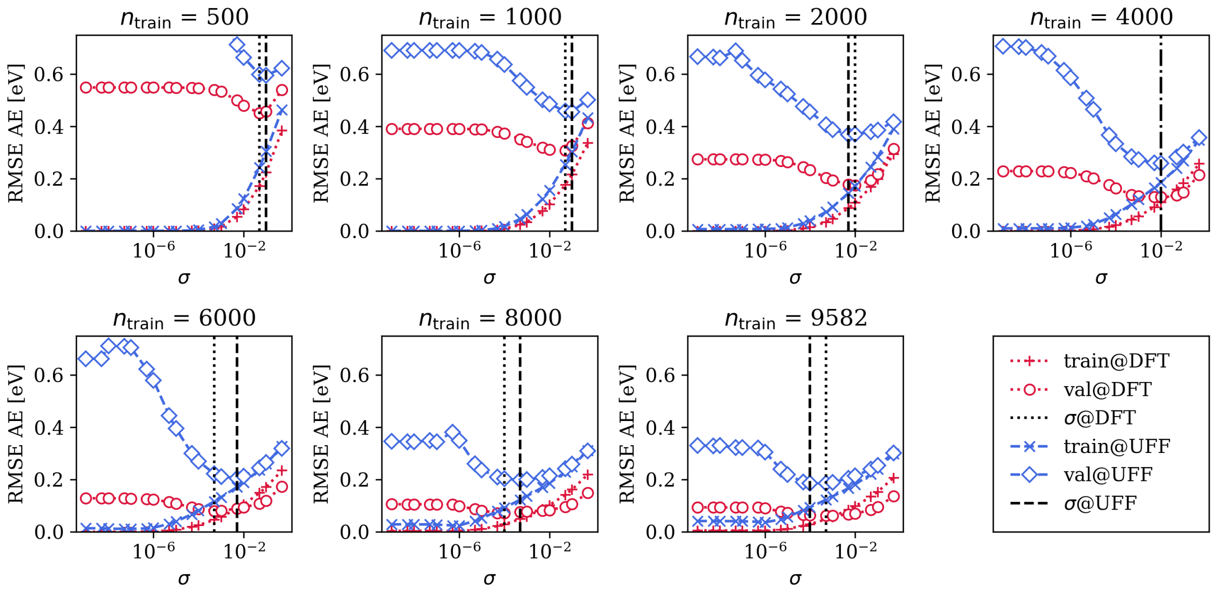

Supplementary Figure 6. Hyperparameter search for K_{ext} FPS int.

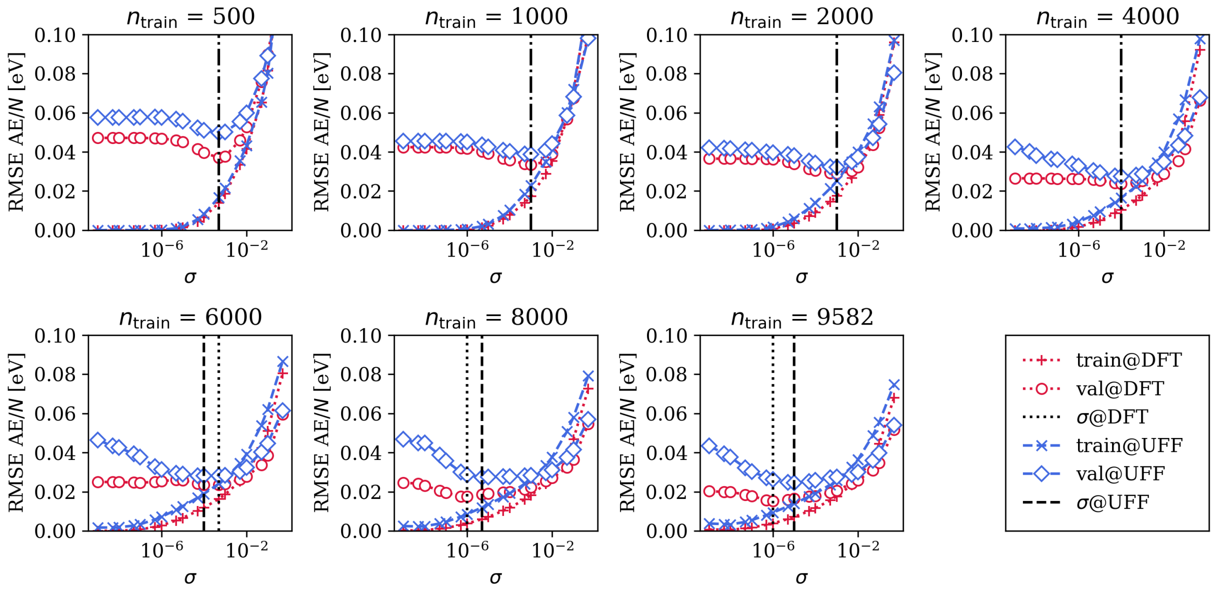

Supplementary Figure 7. Hyperparameter search for K_{int} FPS ext.

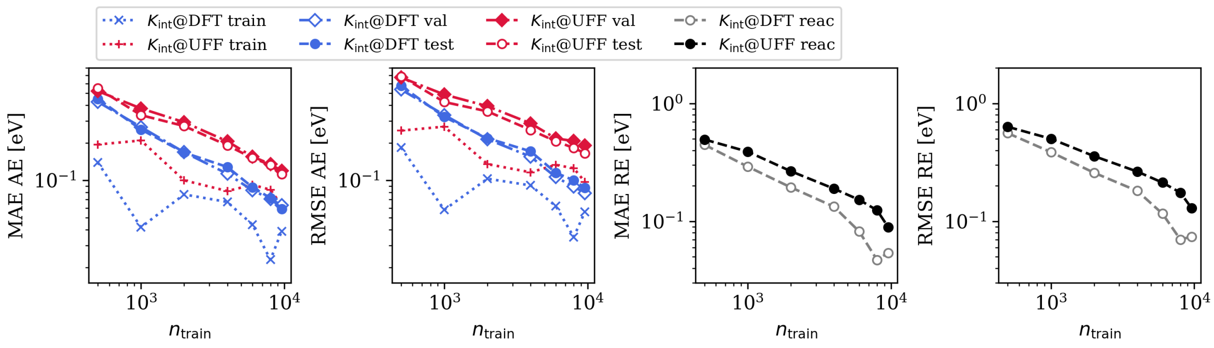

Supplementary Figure 8. Learning curves K_{int} FPS int.

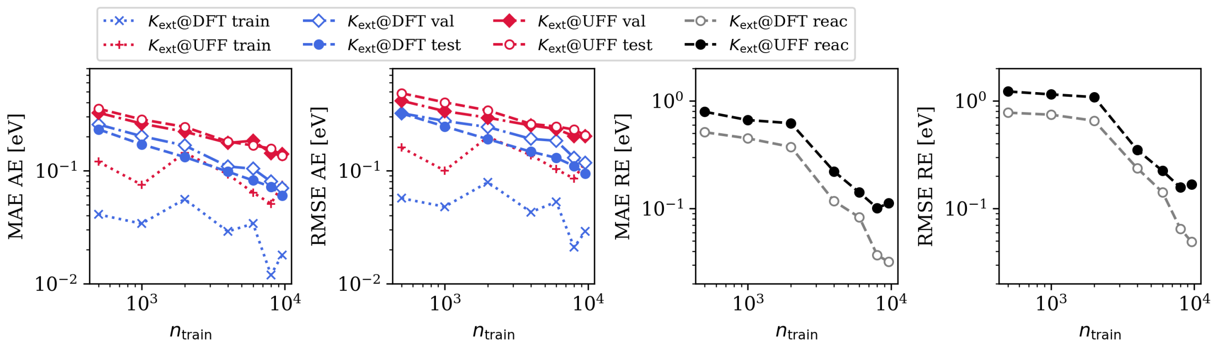

Supplementary Figure 9. Learning curves K_{ext} FPS ext.

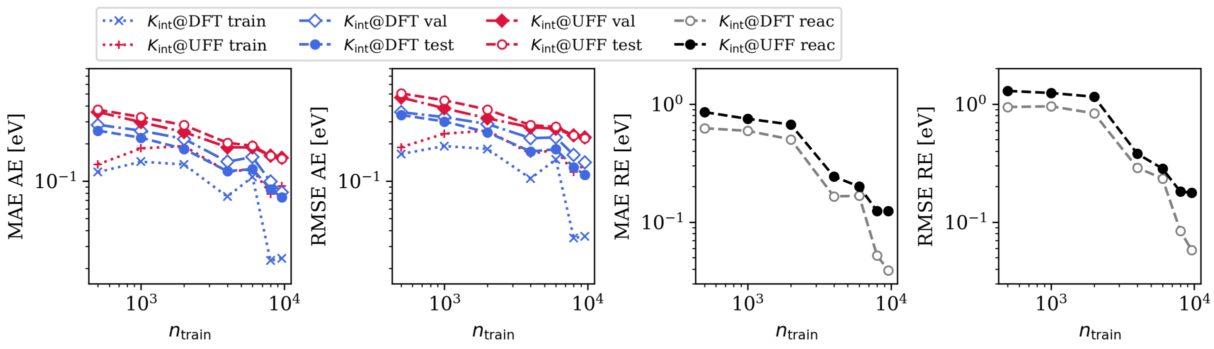

Supplementary Figure 10. Learning curves K_{int} FPS ext.

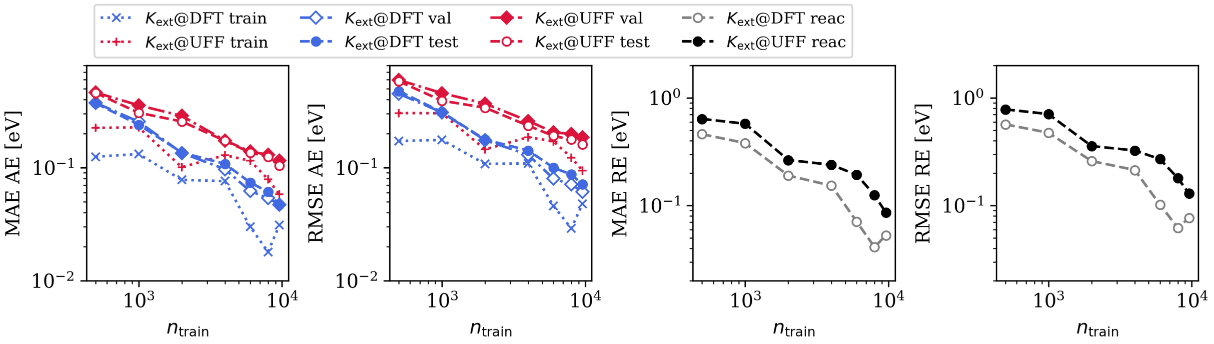

Supplementary Figure 11. Learning curves K_{ext} FPS int.

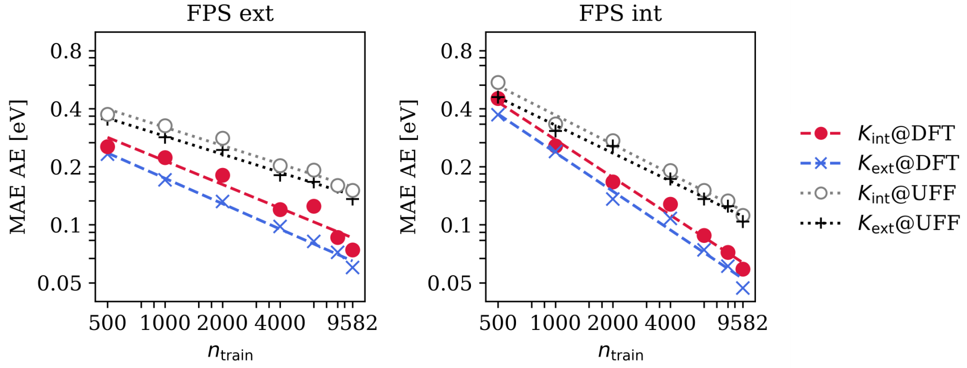

Supplementary Figure 12. Learning curves for atomization energy (AE) predictions (on the test set) using extensive and intensive kernels and DFT and UFF geometries. The two subplots show the results for both FPS splits.

Supplementary Note 5: Timings of ML Model vs. DFT Calculations

A fundamental advantage of using ML is that the predictions for new data points can be made in much less time than the original calculations. To illustrate this we provide the timings for 100 predicitons on random molecules from the Rad-6 database for (1) DFT calculations using computational settings listed in Supplementary Note 1 and (2) a KRR ML model trained on 9582 configurations. More precisely the recorded time for the ML model refers to the calculation of the multisoap average kernel, i.e. the generation of two 9582×100 matrices (K_2 and K_4, see Supplementary Note 2) and the prediction of the 100 molecules using the previously obtained model coefficients α. Unsurprisingly, the KRR model is more than two order of magnitudes faster than a geometry optimization at DFT (PBE0) level.

Supplementary Table 1. Comparison of timings for AE prediction of 100 molecules with the ML model and via full DFT geometry relaxation (at the PBE0+TS level). More details are given in the text.

	DFT	KRR
total time used	949.03 h	1468.19 s
time per molecule	9.49 h	14.68 s

Supplementary Note 6: kPCA

kPCA is a data visualization tool in which huge data sets are intuitively presented and insights into the database are provided. Herein, kPCA is used to have a closer look into the Rad-6 database and visualize similarities and differences between the intensive and extensive kernel (see Supplementary Figures 13, 14).

The location of molecules in the PCA plot is determined by their structural topology. Specifically, PC 1 separates saturated molecules (like hexane) on the left in the PCA plot from very unsaturated ones (like fumaryl) on the right. Simply put, the separation of molecules among PC 1 results in counting hydrogen atoms in the molecules. This is slightly more pronounced in the extensive kernel visible in the colored stripes in Supplementary Figure 13. Furthermore, PC 2 displays the ratio of O / C atoms in the molecule.

The upper and lower right sub-panels in Supplementary Figure 13 show the distribution

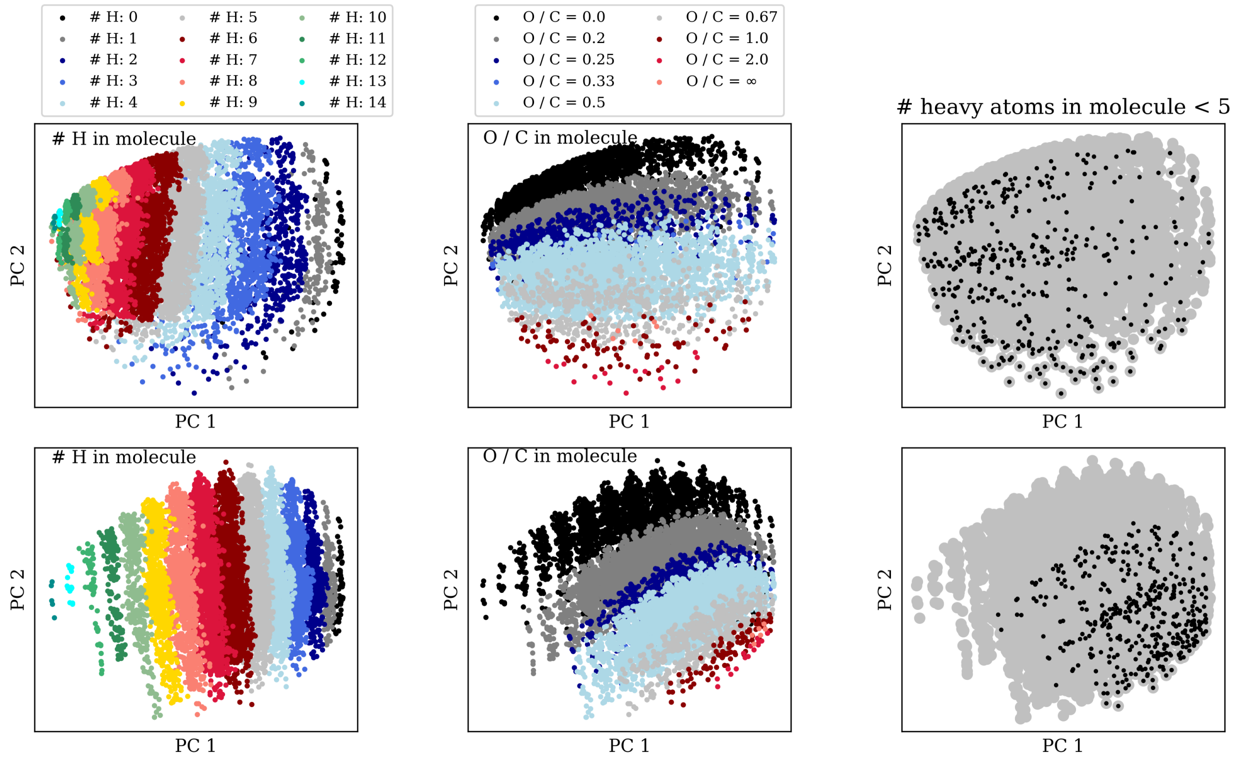

Supplementary Figure 13. kPCA plots of molecules based on the intensive (top) and extensive (bottom) kernel using UFF geometries. Left column: Separation of molecules through PC 1. Colors represent the number of H atoms in a molecule. Middle column: Separation of molecules through PC 2. Colors represent the O / C ratio in a molecule. Right column: Distribution of small molecules with maximum 4 heavy atoms in a molecule.

of small molecules located in the database. We denote molecules with a maximum number of 4 heavy atoms (i.e. non H-atoms) as small molecules. These account for around 4 % of the database. While the small molecules are distributed over the whole space in the plot of K_{int}, for K_{ext} they are bounded on the bottom right. This picture illustrates why small molecules are selected relatively late in FPS with the extensive kernel, because the distances among them are relatively close.

Supplementary Figure 14 is an extension of Fig. 3 in the main text. The plot shows the kPCA for the extensive (bottom) and intensive (top) kernels colored by the predicted atomization energies and atomization energies per atom for ML models with 1000 training configurations, respectively. Additionally, the differences between the ML models and the reference DFT calculated energies are displayed on the right. K_{ext} shows higher errors on

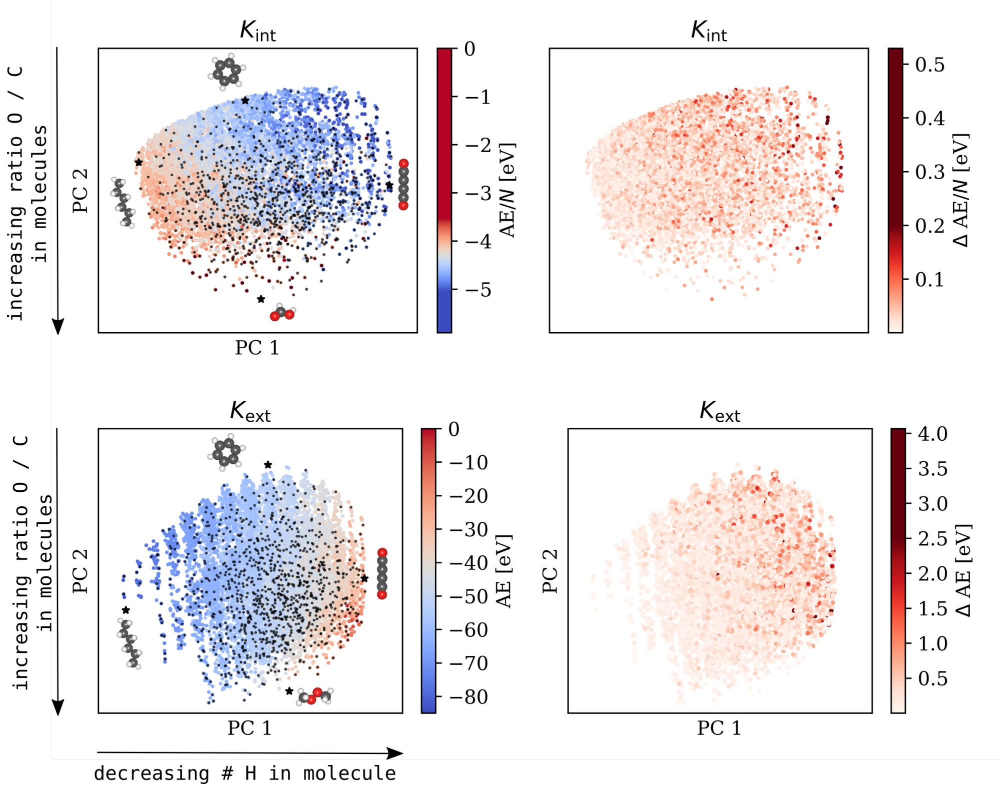

Supplementary Figure 14. kPCA plot of molecules based on the intensive (top) and extensive (bottom) kernel using UFF geometries: Points are colored according to the predicted atomization energy per atom (top left) and the predicted total atomization energy (bottom left) using ML models with 1000 training points. The absolute differences between the ML models and DFT reference values are shown on the top right picture for K_{int} and on the bottom right for K_{ext}. Small black dots indicated training structures. The arrows provide a qualitative interpretation of the principal component axes.

the predictions on the right half of the plot where especially small molecules are located. This illustrates again the poor performance of the extensive kernel in predicting reaction energies. A precise description of small molecules is crucial for calculating reaction energies, since they represent important hubs in the reaction network.

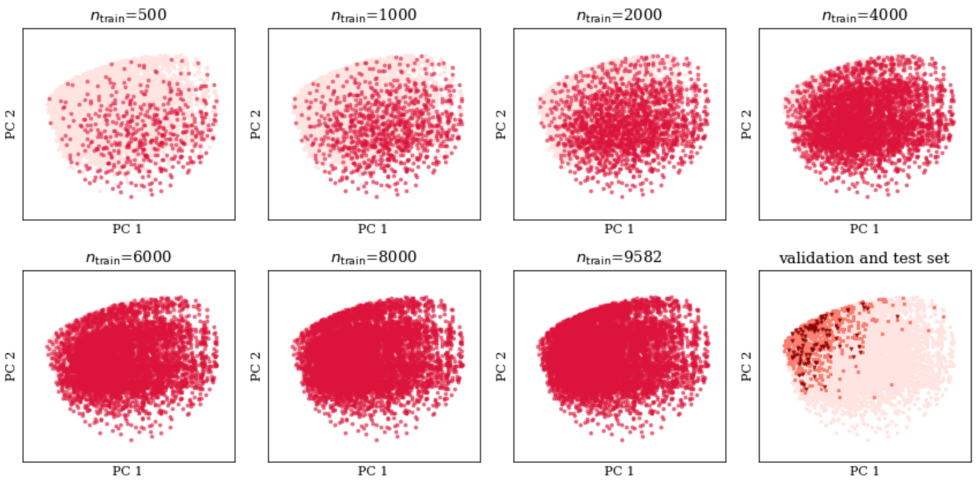

Supplementary Figure 15. kPCA plot for the intensive kernel and an intensive FPS split. The individual subplots show the distribution of training configurations with different training set sizes. The bottom right panel shows the distribution of validation (triangles) and test set (squares).

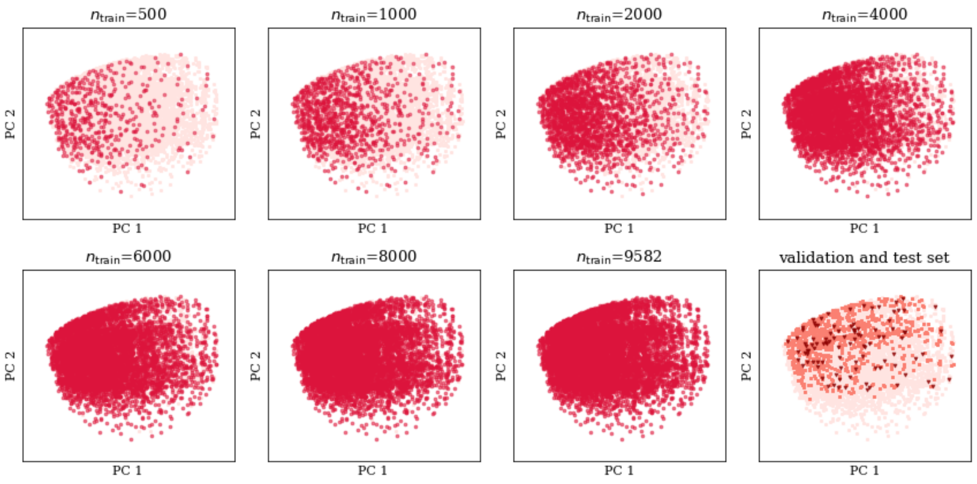

Supplementary Figure 16. kPCA plot for the intensive kernel and an extensive FPS split showing the distribution of the training, validation and test set configurations (see Supplementary Figure 15).

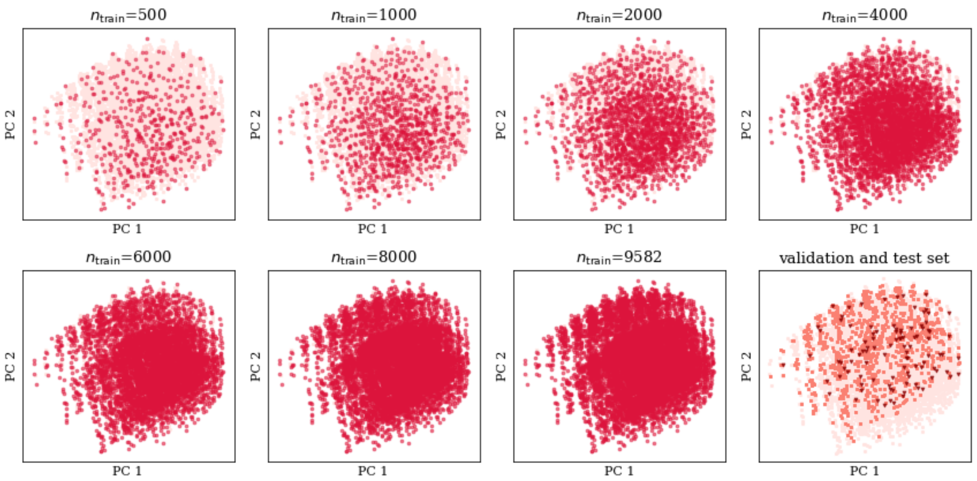

Supplementary Figure 17. kPCA plot for the extensive kernel and an extensive FPS split showing the distribution of the training, validation and test set configurations (see Supplementary Figure 15).

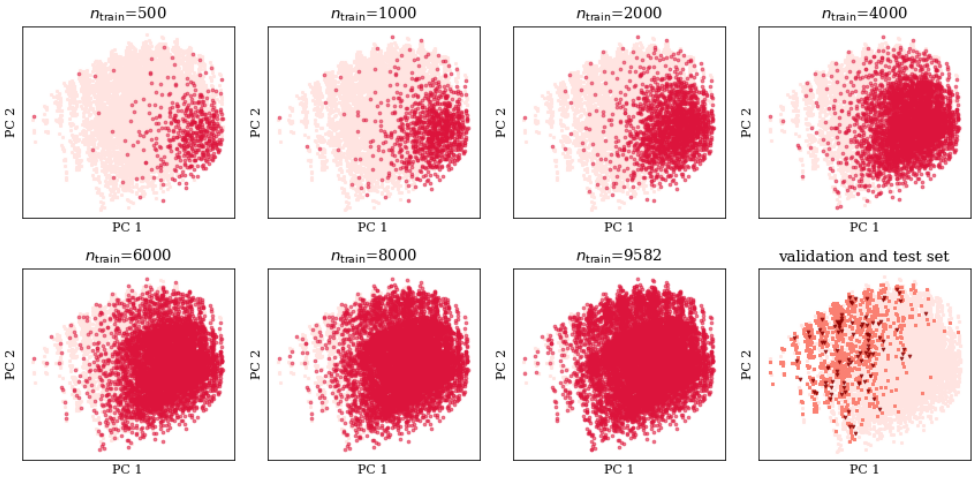

Supplementary Figure 18. kPCA plot for the extensive kernel and an intensive FPS split showing the distribution of the training, validation and test set configurations (see Supplementary Figure 15).

Supplementary Note 7: σ-Scaling

As discussed in the main text, for real applications using forcefield or semiempirical instead of DFT geometries is inevitable. The description of molecular geometries with UFF can be of varying quality for different molecules, which implies that there is not a constant level of noise on the reference data (see Supplementary Figure 19). To this end, Bartók et al.[10] suggested to weight training structures so that the ML model naturally assumes higher uncertainties for configurations that have poor geometries. In their work they quantify the difference between high and low level structures as the root mean square deviation (RMSD) d and scale the regularization parameter σ to be proportional to the factor $f = \exp(\frac{d^2}{\lambda})$. By this a new hyperparameter λ arises that has to be determined empirically. To estimate the range of reasonable parameters we plot the scaling factor f as a function of λ using the maximum and mean RMSD in the database (see Supplementary Figure 20). The plot shows a huge deviation between the scaling factors, especially for small λ. In this case structures with a large RMSD are scaled by 4-17 orders of magnitude and structures with an average RMSD by around 1 order of magnitude for the three lowest λ values.

The results of learning the atomization energies with and without σ-scaling for K_{ext} and K_{int} with both FPS splits are shown in Supplementary Figure 21. We found that σ-scaling does not effect the prediction of AEs using the intensive FPS split. For every point in the learning curve the RMSE for validation and test set remains the same and the λ values assume one of the highest values resulting in a scaling factor of 1.

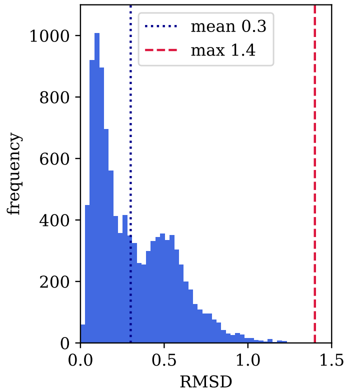

Supplementary Figure 19. Histogram of RMSD values. Average (dotted) and maximum RMSD (dashed) are indicated.

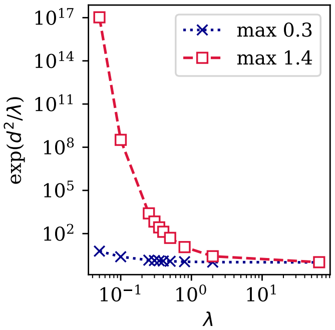

Supplementary Figure 20. Illustrative scaling values for the diagonal elements using the maximum RMSD $d = 1.4$ Å and average RMSD $d = 0.3$ Å. The labels represent the used λ values in the grid search.

In contrast, the results change somewhat for the predictions using the extensive FPS split (left subplots in Supplementary Figure 21). In these cases, σ-scaling lowers the error of the validation set, but increases the RMSE in the test set for both the extensive and the intensive kernel and thus leads to some degree of over-fitting.

To conclude, an improvement of the predictions for AE using the RMSD of UFF and DFT training configurations to scale the regularization parameter was not successful. This is likely due to the different and poor quality of UFF geometries of open-shell structures.

In this work the RMSD values are calculated with the code `rmsd` obtained from GitHub.[24,25] Since the molecules for the UFF and DFT geometry optimization are created from the same smile string, no reordering was applied.

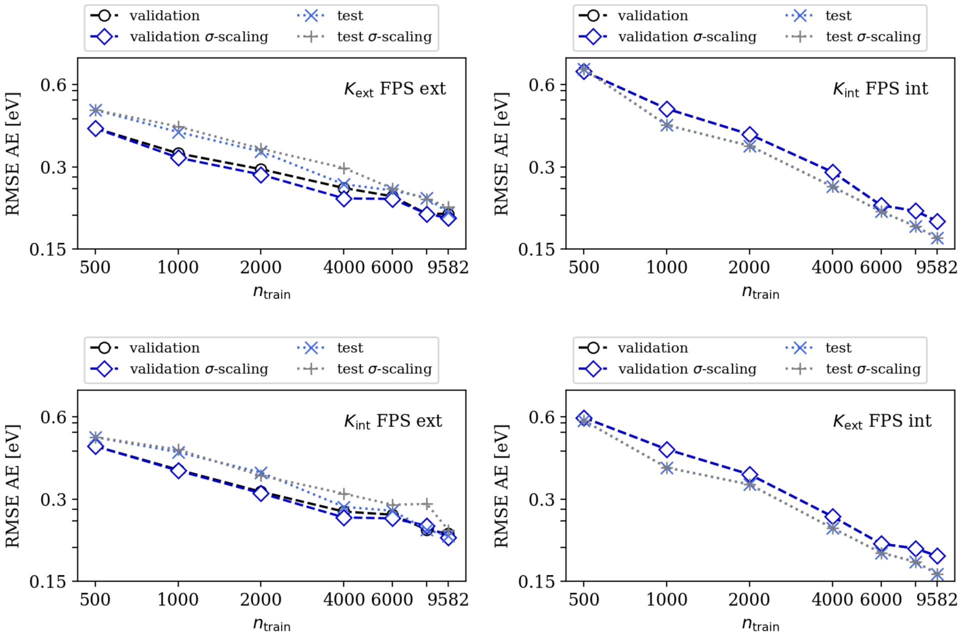

Supplementary Figure 21. Learning curves of AE predictions for validation and test structures with and without σ-scaling using the extensive and intensive kernels with both FPS splits. The RMSE is displayed because the hyperparameter are selected according to the minimum RMSE of the validation set.

Supplementary Note 8: Learning RE with UFF Geometries

Supplementary Figure 22 shows the correlation plots between the predicted atomization energies and reaction energies for DFT and UFF geometries. Comparable to the learning of AE, trends for RE with UFF geometries are similar to those with DFT, but with an higher MAE.

Supplementary Note 9: Training Set Selection with Random Sampling

In this section we show the performance for the prediction of AE and RE using random sampling for training set selection in contrast to the farthest point sampling used in the main manuscript. To this end we generated a randomly chosen sequence of up to 9582 training, 100 validation (for hyperparameter optimization) and 1030 test configurations. This split is applied to the predictions of atomization energies and corresponding reaction energies of Rad-6-RE using the extensive and the intensive kernels. kPCA plots illustrating the random

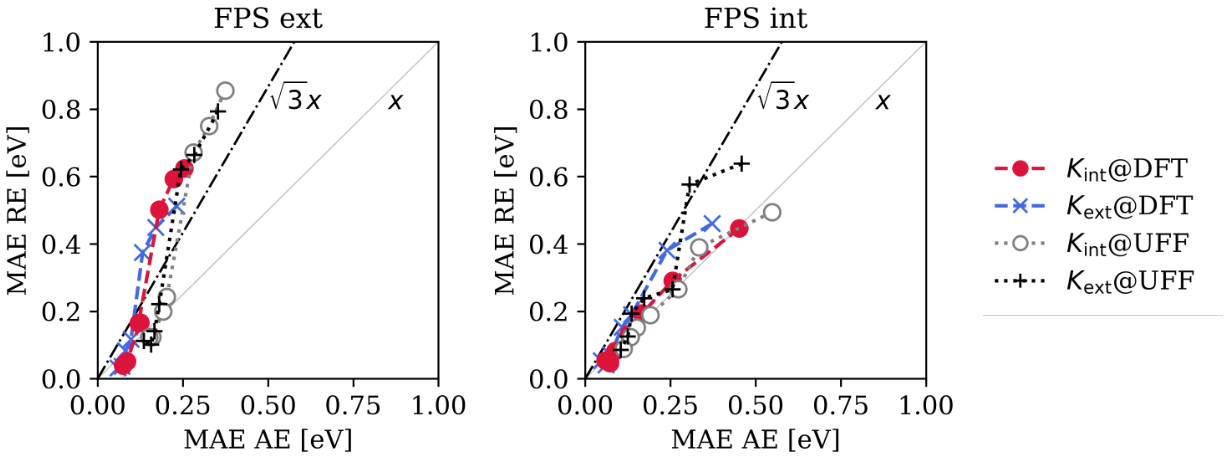

Supplementary Figure 22. Mean absolute errors (MAEs) for AE and RE predictions using DFT (dashed lines) and UFF (dotted lines) geometries and the extensive and intensive kernels described in the manuscript. Multiple points for each model represent the different training set sizes shown in Supplementary Figure 12.

sets are shown in Supplementary Figures 23 and 24.

Hyperparameter search: The hyperparameter search was performed as described in Supplementary Note 3 (see also Supplementary Figures 25 and 27). However, an exception was made in the case of the intensive kernel. Here, only 99 molecules were used in the validation set to determine the regularisation parameter σ. This is because large errors for the carbon dimer lead to a poor choice of σ in this case (i.e. the models were severely underfitted). This illustrates the dangers of pure random sampling: C_2 has low similarity with all other molecules in the dataset and should therefore be included in the training set (see also Supplementary Figure 26).

Learning curves: The learning curves are analogous to Supplementary Figures 8-11 but use training sets from random sampling.

Learning atomization energies: Supplementary Figure 30 shows the results of the atomization energy predictions for random sampling (right subplot) together with both FPS splits for the intensive and extensive kernels. The general trends with respect to kernel selection and the effect of UFF vs. DFT geometries are the same in all cases. However, the prediction errors for large training sets are somewhat larger in the case of random sampling, though it is competitive for small training sets.

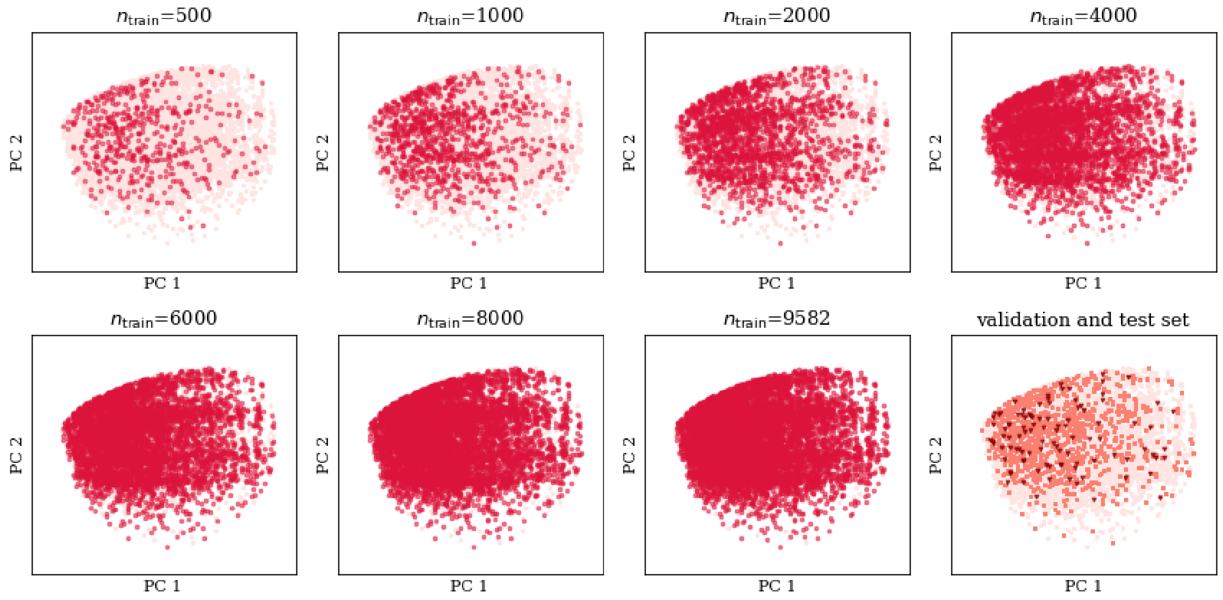

Supplementary Figure 23. kPCA plot for the intensive kernel and a random training set sampling. The individual subplots show the distribution of training configurations with different training set sizes. The bottom right panel shows the distribution of validation (triangles) and test set (squares).

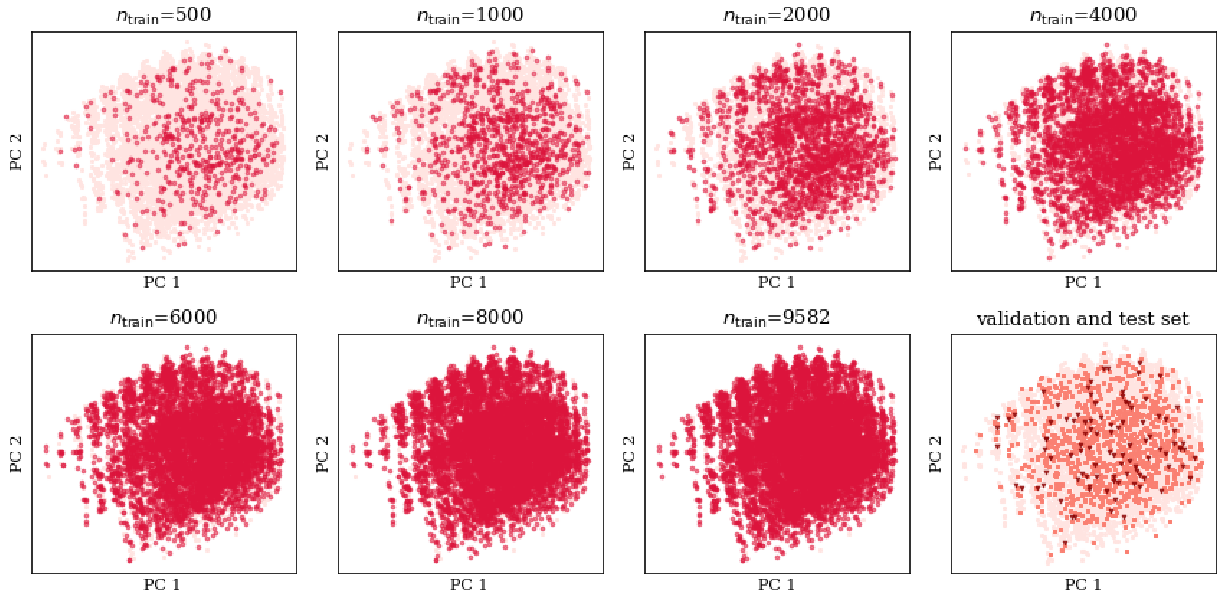

Supplementary Figure 24. Same plot as Supplementary Figure 23 but for the extensive kernel with random training set selection.

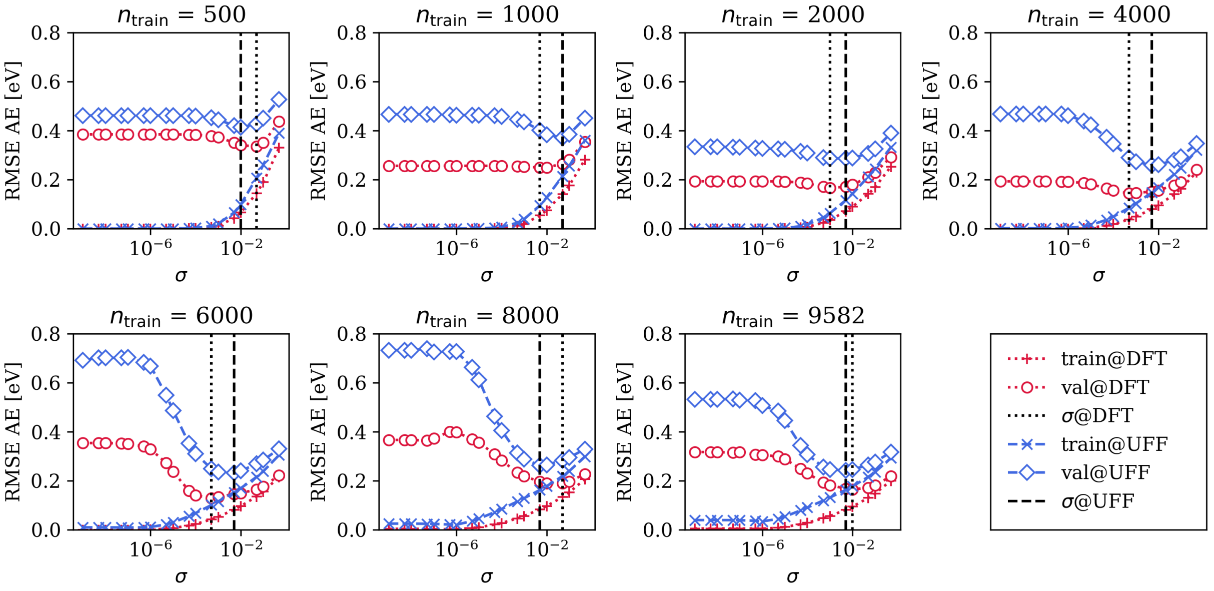

Supplementary Figure 25. Hyperparameter search for $K_{\rm ext}$ and random sampling.

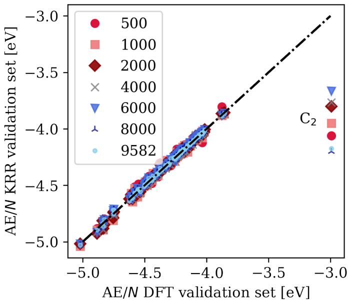

Supplementary Figure 26. Correlation plot of DFT calculated and predicted AE/N for the validation set with the intensive kernel using random sampling and different training set sizes.

Learning reaction energies: In this section we compare the results of random training set selection with farthest point sampling for the prediction of reaction energies in the Rad-6-RE network (see Supplementary Figure 31). We see that for small training set sizes, random sampling performs drastically worse for the predictions of reaction energies. Similar to what is observed for the extensive FPS split, this is attributed to large errors for essential 'hub' molecules, which are absent from the training set. This is only mitigated for the larger training sets, which approach the FPS sets (though still displaying larger MAEs).

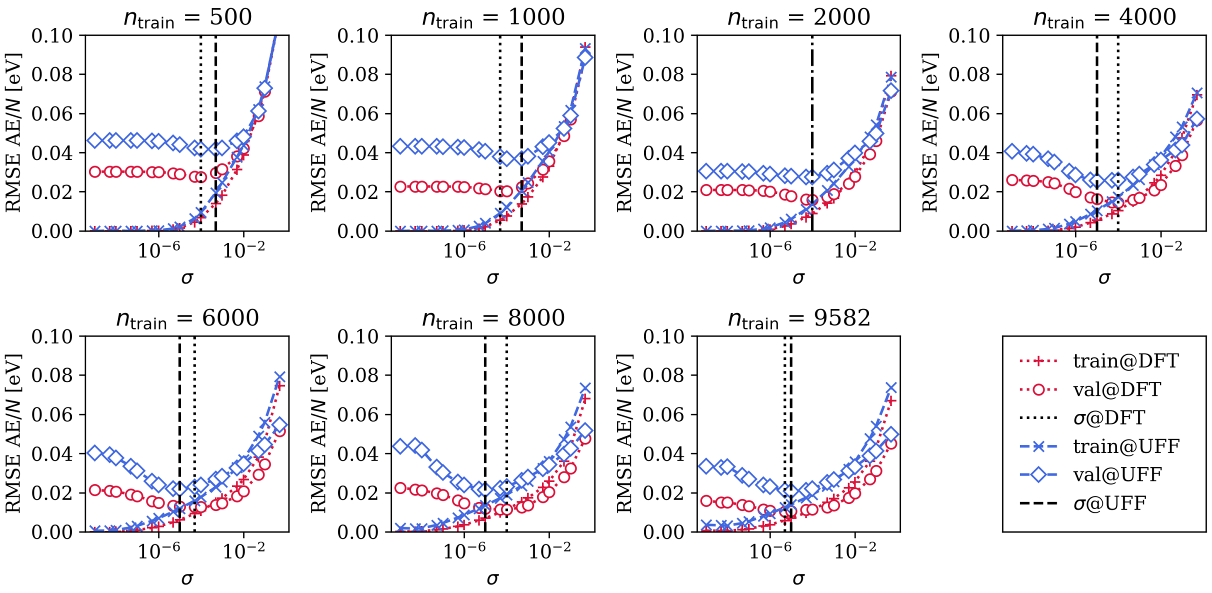

Supplementary Figure 27. Hyperparameter search for K_{int} and random sampling.

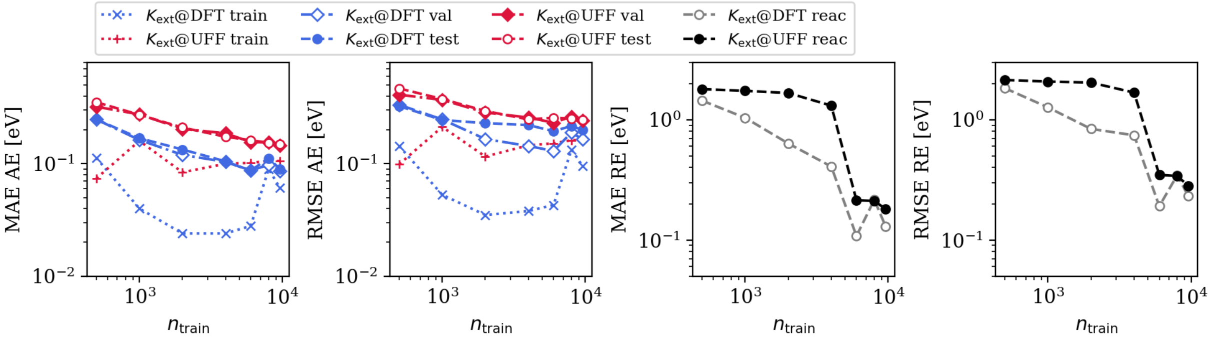

Supplementary Figure 28. Learning curves for K_{ext} and random sampling.

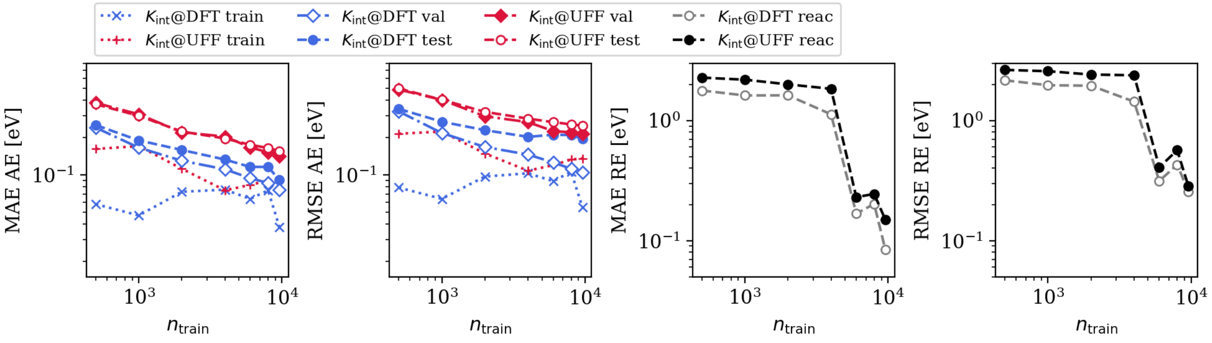

Supplementary Figure 29. Learning curves for K_{int} and random sampling.

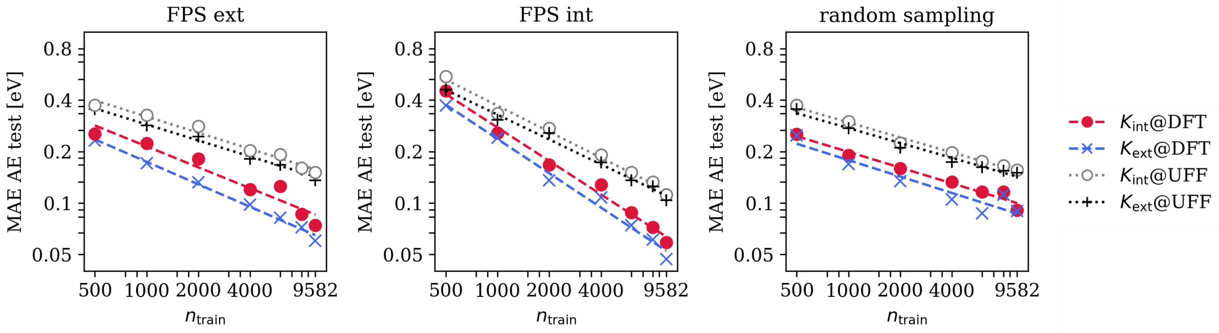

Supplementary Figure 30. Comparison of learning curves for atomization energy (AE) predictions using extensive and intensive kernels for both DFT and UFF geometries. The three subplots show the results for the entensive and intensive FPS splits as well as for random sampling.

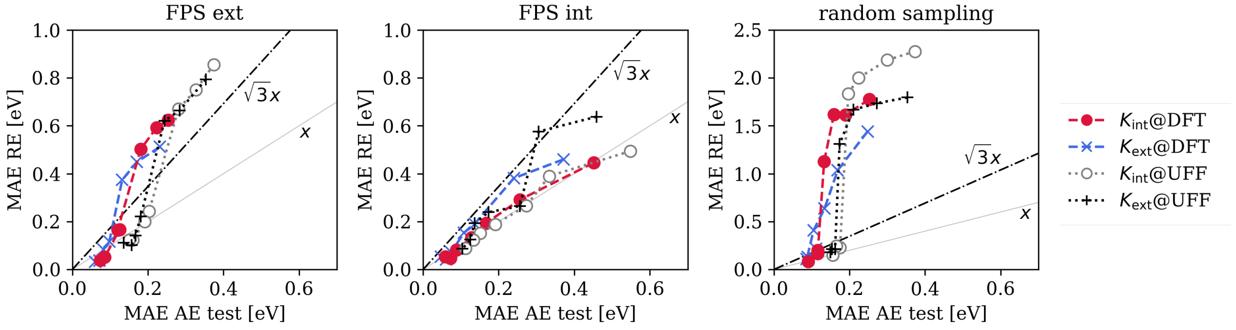

Supplementary Figure 31. Mean absolute errors (MAEs) for AE and RE predictions using DFT (dashed lines) and UFF (dotted lines) geometries and the extensive and intensive kernels for both FPS splits and random sampling. Multiple points for each model represent the different training set sizes shown in Supplementary Figure 30.

Supplementary Note 10: Comparison Rad-6 and Rad-6-BS

As discussed in the manuscript, the choice of reference spin-states taken for Rad-6 is somewhat arbitrary and may not be ideal for every application. Nonetheless, we expect the ML methodology developed herein to be equally applicable to reference data with different choices in spin-states. In this light, it is instructive compare the results from the main manuscript with models trained on the Rad-6-BS database (for computational details see Supplementary Note 1.)

The corresponding hyperparameter searches, learning curves and final results are shown

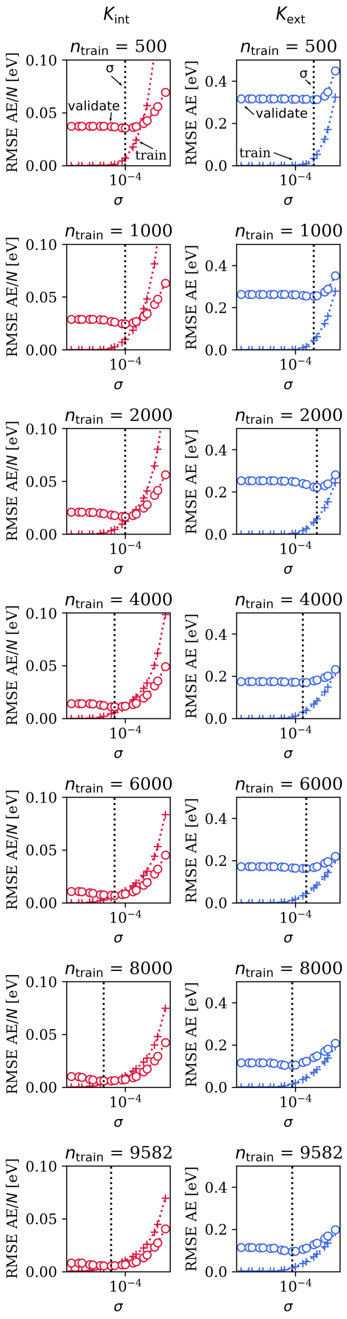

Supplementary Figure 32. Hyperparameter search for ML models of the Rad-6-BS database. The panels show the hyperparameter surfaces for the intensive (left) and extensive (right) kernel.

in Supplementary Figures 32-33 . These results are obtained with K_{int} and K_{ext} and the corresponding FPS split. As expected the differences in the reference methods between Rad-6 and Rad-6-BS do not significantly affect the performance of the ML models for AE and RE prediction.

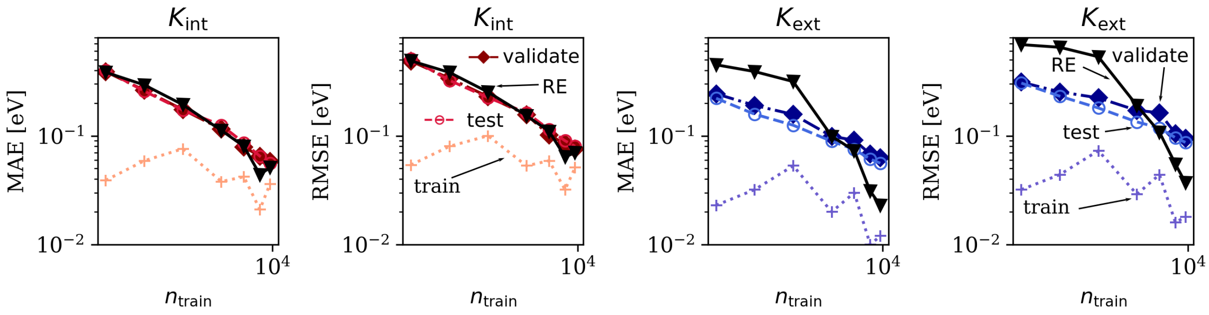

Supplementary Figure 33. Learning curves for Rad-6-BS predictions showing the MAE and RMSE for the intensive and extensive kernel. Dotted positive signs are the AE errors of the training sets, solid diamonds are the AE errors of the validation sets, dashed circles are the AE errors of the test sets and solid triangles are the errors of the reaction energies.

Supplementary Note 11: Microkinetic Simulation

In the main text, we explore a realistic reaction network consisting of 21,392 reactions using an approximate microkinetic simulation. This network contains bond-breaking, transfer and rearrangement reactions of the general form:

$$A + B \rightleftharpoons C + D,$$

where molecules B and/or D can be 'empty' placeholders for bond-breaking and rearrangement reactions.[2]

The kinetics of this reaction network are governed by differential equations of the form:

$$\frac{d\theta_A}{dt} = -\sum_{B,CD} 2^{\delta_{AB}} \theta_A \theta_B k_{AB}^{CD} + \sum_{CD,B} 2^{\delta_{AB}} \theta_C \theta_D k_{CD}^{AB},$$

where θ_A is the concentration of molecule A, k_{AB}^{CD} is the rate constant for the reaction $A + B \rightarrow C + D$. Note that the first sum is over all elementary reactions that consume A, and the second sum is over the corresponding reverse reactions, where A is formed.

The term $\theta_A \theta_B k_{AB}^{CD}$ corresponds to the current rate of a given reaction, r_{AB}^{CD}. In other words, the rate depends on the concentration of the educts and the rate constant k_{AB}^{CD}, which is in turn proportional to the reaction energy and the activation energy. As mentioned in the main text, all activation energies are assumed to be identical. We can then compute the rate constants from transition state theory *via*:

$$k_{AB}^{CD} = e^{\frac{-\Delta E}{k_B T}}$$

Here, the energy difference ΔE is the reaction energy plus the activation energy for an endothermic reaction and the activation energy for an exothermic reaction. Under these circumstances, the actual value of the activation energy is not important (it is chosen to be 0.3 eV), and only changes the arbitrary time unit of the simulation. Similarly, we choose a constant pre-exponential factor of 1 for all reactions.

The simulation is initialized with equal concentrations of CH_4 and O_2, all other concentrations set to 0. At the beginning of the simulation, all rates are thus also 0, except for reactions involving CH_4 and O_2. We then propagate the differential equations specified above using a third-order Runge-Kutta integrator.[26] As the concentrations are updated, more rates become larger than zero. The subgraphs shown in the main manuscript show all reactions with non-zero rates at a given simulation time.

Supplementary Note 12: Validation of Out-Of-Sample Predictions

As discussed in the main manuscript, the reaction network used in the microkinetic analysis contains several systems that are not included in Rad-6, and thus represent a true out-of-sample application of the ML model. To evaluate the quality of these predictions, DFT calculations were preformed on these out-of-sample systems. Unfortunately, these systems are missing from Rad-6 because they either decomposed upon geometry relaxation or had SCF convergence issues in the original high-throughput simulations for the database. We were, however, able to obtain single-point DFT energies on frozen UFF geometries (DFT@UFF, same computational settings as for Rad-6) for all but one of these systems.

In Supplementary Figure 34, correlation plots for DFT@UFF, DFT@DFT and ML predicted AEs are shown (with the out-of-sample systems highlighted in blue). As expected, the ML and DFT@DFT values display an excellent correlation. Meanwhile, both of these approaches consistently predict more negative AEs than the DFT@UFF approach, since the latter is missing geometry relaxation effects. Importantly, this is also the case for the out-of-sample predictions, meaning that the ML model can be used to estimate relaxation effects even when DFT relaxations are not available.

Overall, there is also a good correlation between the DFT@UFF values and the ML predictions, with R^2 =0.994. To quantify the magnitude of geometry relaxation effects, we calculate the mean error (ME) between DFT@UFF and ML, in addition to the MAE. We

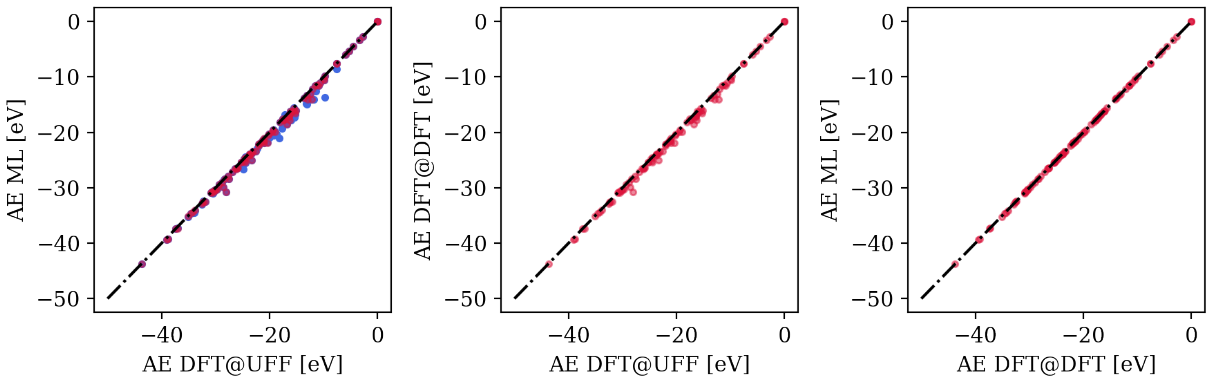

Supplementary Figure 34. Correlation plots for predicted atomization energies. Left: ML model (used in main text) vs. single point DFT calculations at UFF geometries (DFT@UFF). Middle: Optimized DFT calculations (DFT@DFT) vs. DFT@UFF. Right: ML model vs. DFT@DFT. Note that the left panel contains the additional out-of-sample data points (highlighted in blue, see text for details).

find that the ME and MAE are nearly identical (ca. 0.6 eV, see Supplementary Table 2), confirming the systematic nature of the deviation. For comparison, the corresponding DFT@DFT values are also shown, again with identical ME and MAE. Note that the deviations relative to DFT@UFF are not identical for ML and DFT@DFT because the ML comparison includes more systems (the out-of-sample set). Taken as a whole, these observations provide a strong indication that our ML model predicts reasonable AEs for the out-of-sample molecules in the network.

This data is also used to verify the reaction energies that go into the microkinetic simulations, as shown in Supplementary Figure 35. Again, we find a good correlation between our ML model and the DFT@UFF calculations, with some scatter. Importantly, similar correlation and scatter are observed when comparing DFT@DFT and DFT@UFF, confirming the high quality of the ML predictions.

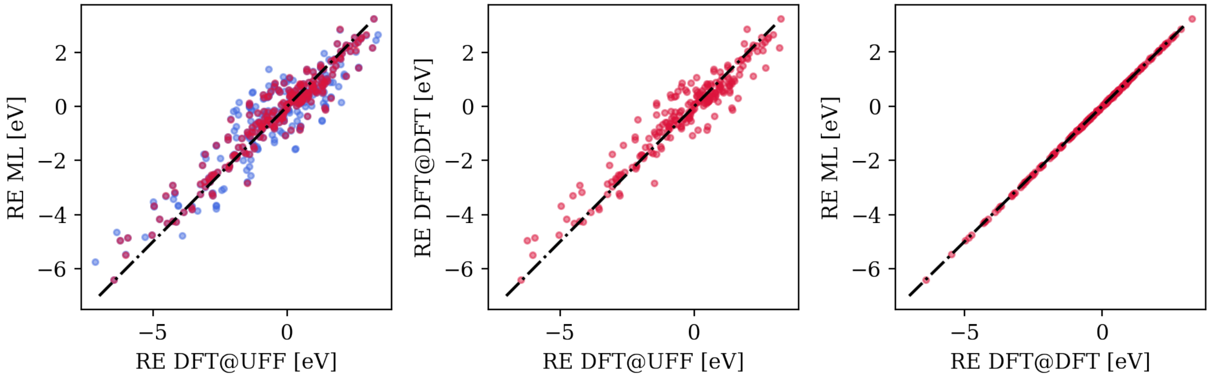

Supplementary Figure 35. Correlation plots for reaction energies. Labels are analogous to Supplementary Figure 34. Shown reaction energies are from the reduced network at t=128 (see manuscript for details).

Supplementary Table 2. Summary of statistics (MAE, ME and R^2) pertaining to the plots in Supplementary Figures 34 and 35.

	MAE AE [eV]	ME AE [eV]	R^2	N
DFT@UFF - ML	0.606	0.599	0.994	130
DFT@UFF - DFT@DFT	0.414	0.414	0.997	101
DFT@DFT - ML	0.022	0.0005	1.000	101
	MAE RE [eV]	ME RE [eV]	R^2	N
DFT@UFF - ML	0.572	-0.044	0.840	365
DFT@UFF - DFT@DFT	0.420	-0.074	0.910	225
DFT@DFT - ML	0.009	0.0004	1.000	225

SUPPLEMENTARY REFERENCES

[1]Weininger, D. *J. Chem. Inf. Comput. Sci.* **1988**, *28*, 31–36.

[2]Margraf, J. T.; Reuter, K. *ACS Omega* **2019**, *4*, 3370–3379.

[3]RDKit: Open-source cheminformatics. `http://www.rdkit.org`.

[4]Rappe, A. K.; Casewit, C. J.; Colwell, K. S.; Goddard, W. A.; Skiff, W. M. *J. Am. Chem. Soc.* **1992**, *114*, 10024–10035.

[5]Blum, V.; Gehrke, R.; Hanke, F.; Havu, P.; Havu, V.; Ren, X.; Reuter, K.; Scheffler, M.

Comput. Phys. Commun. **2009**, *180*, 2175 – 2196.

(6) Zhang, I. Y.; Ren, X.; Rinke, P.; Blum, V.; Scheffler, M. *New J. Phys.* **2013**, *15*, 123033.

(7) Adamo, C.; Barone, V. *J. Chem. Phys.* **1999**, *110*, 6158–6170.

(8) Tkatchenko, A.; DiStasio, R. A.; Car, R.; Scheffler, M. *Phys. Rev. Lett.* **2012**, *108*, 236402.

(9) Rupp, M. *Int. J. Quantum Chem.* **2015**, *115*, 1058–1073.

(10) Bartók, A. P.; De, S.; Poelking, C.; Bernstein, N.; Kermode, J. R.; Csányi, G.; Ceriotti, M. *Sci. Adv.* **2017**, *3*, e1701816.

(11) Neese, F. *WIREs Comput. Mol. Sci.* **2018**, *8*, e1327.

(12) Zhang, Y.; Yang, W. *Phys. Rev. Lett.* **1998**, *80*, 890.

(13) Vaucher, A. C.; Reiher, M. *J. Chem. Theory Comput.* **2017**, *13*, 1219–1228.

(14) Deringer, V. L.; Csányi, G. *Phys. Rev. B* **2017**, *95*, 094203.

(15) Szlachta, W. J.; Bartók, A. P.; Csányi, G. *Phys. Rev. B* **2014**, *90*, 104108.

(16) Cliffe, M. J.; Bartók, A. P.; Kerber, R. N.; Grey, C. P.; Csányi, G.; Goodwin, A. L. *Phys. Rev. B* **2017**, *95*, 224108.

(17) Bartók, A. P.; Kondor, R.; Csányi, G. *Phys. Rev. B* **2013**, *87*, 184115.

(18) De, S.; Bartók, A. P.; Csányi, G.; Ceriotti, M. *Phys. Chem. Chem. Phys.* **2016**, *18*, 13754–13769.

(19) `https://github.com/libAtoms/QUIP`.

(20) `https://github.com/simonwengert/mltools.git`.

(21) Ceriotti, M.; Willatt, M. J.; Csányi, G. Machine-learning of atomic-scale properties based on physical principles. `https://arxiv.org/abs/2001.11696`.

(22) Rasmussen, C.; Williams, C. *Gaussian Processes for Machine Learning*; MIT Press, 2006.

(23) Schölkopf, B.; Smola, A.; Müller, K.-R. *Neural Comput.* **1998**, *10*, 1299–1319.

(24) Kabsch, W. *Acta Cryst. A* **1976**, *32*, 922–923.

(25) `https://github.com/charnley/rmsd`.

(26) Bogacki, P.; Shampine, L. *Applied Mathematics Letters* **1989**, *2*, 321 – 325.
