# Supplementary material for: Machine learning in chemical reaction space
Source: Nat Commun. 2020 Oct 30;11:5505. doi: 10.1038/s41467-020-19267-x (PMC7603480; doi:10.1038/s41467-020-19267-x)
Supplement: Supplementary file 2 — Description of Additional Supplementary Files [file 41467_2020_19267_MOESM2_ESM.pdf]

Description of Additional Supplementary Files

File Name: Supplementary Data 1
Description: DFT reference data, molecular geometries, regression model coefficients, farthest point sampling splits.
